# Magnetic Anisotropy Trends along a Full 4f-Series: The *f*^*n*+7^ Effect

**DOI:** 10.1021/jacs.1c02502

**Published:** 2021-05-24

**Authors:** Matteo Briganti, Eva Lucaccini, Laura Chelazzi, Samuele Ciattini, Lorenzo Sorace, Roberta Sessoli, Federico Totti, Mauro Perfetti

**Affiliations:** † Department of Chemistry “U. Schiff”, University of Florence Via della Lastruccia 3-13, Sesto Fiorentino (FI) 50019, Italy; ‡ Center of Crystallography, University of Florence, Via della Lastruccia 3, Sesto Fiorentino (FI) 50019, Italy

## Abstract

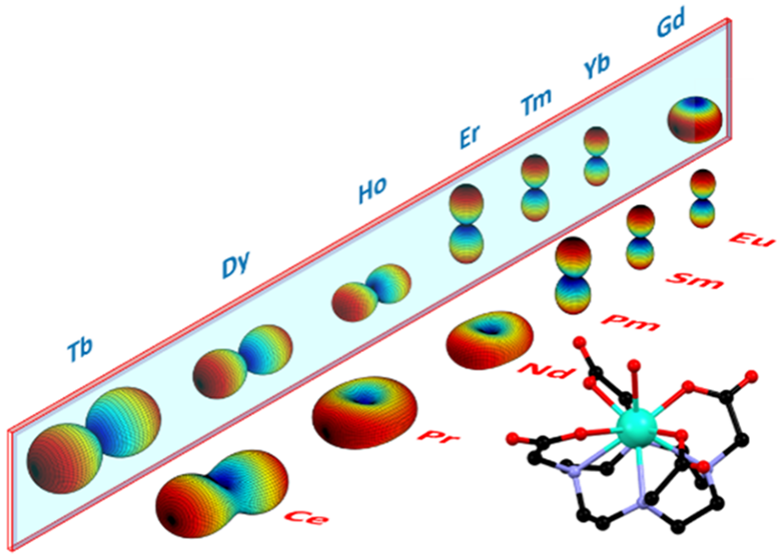

The combined experimental and computational study of the 13 magnetic complexes belonging to the Na[LnDOTA(H_2_O)] (H_4_DOTA = tetraazacyclododecane-*N*,*N*′,*N*″,*N*‴-tetraacetic acid and Ln = Ce–Yb) family allowed us to identify a new trend: the orientation of the magnetic anisotropy tensors of derivatives differing by seven *f* electrons practically coincide. We name this trend the *f*^*n*+7^ effect. Experiments and theory fully agree on the match between the magnetic reference frames (e.g., the easy, intermediate, and hard direction). The shape of the magnetic anisotropy of some couples of ions differing by seven f electrons might seem instead different at first look, but our analysis explains a hidden similarity. We thus pave the way toward a reliable predictivity of the magnetic anisotropy of lanthanide complexes with a consequent reduced need of computational and synthetical efforts. We also offer a way to gain information on ions with a relatively small total angular momentum (i.e., Sm^3+^ and Eu^3+^) and on the radioactive Pm^3+^, which are difficult to investigate experimentally.

## Introduction

Deep comprehension of the factors determining magnetic anisotropy is the key for improving the performances of lanthanide-based magnetic materials, already used in a wide range of different fields ranging from biochemistry^1^ to medicine,^2^ from solid state physics^3^ to cryogenics.^4,5^ Synthetic chemists are nowadays able to tailor ligands with a suitable number and position of donor atoms to enhance the magnetic anisotropy of the magnetic ion and avoid interactions that can lead to unfavorable effects on magnetic properties.^6−9^ The quest to find periodic correlations and establish whether a theoretical model is robust enough to predict properties of the entire lanthanide series makes the collection of data on several isostructural Ln complexes highly desirable. While magnetic studies on the second half of the series (Tb to Yb) are common, publications dealing with both heavy and light lanthanides are extremely rich in information but rare.^10−16^ Even if this might seem astonishing, to the best of our knowledge there are no studies on magnetic properties of complete series (all 13 magnetic elements, from Ce to Yb) of mononuclear Ln molecular compounds.

Some simple models based on the electronic density of the 4f shell and on electrostatic interaction to predict the magnetic anisotropy along the Ln series have been proposed and largely followed.^17−19^ However, there are several examples of compounds that need a more accurate description to obtain a correct prediction of the shape and strength of magnetic anisotropy.^20−22^ One such example is the [Ln(DOTA)(H_2_O)]^−^ series. The DOTA^4–^ ligand forms stable complexes with a large number of metal ions.^23^ Among them, the high affinity for Ln leads to a remarkable kinetic and thermodynamic stability in solution.^24,25^ Several studies on their conformational equilibrium in solution are also present in the literature.^26,27^ The DOTA^4–^ ligand is particularly useful in the field of magnetic resonance imaging (MRI), due to its peculiar chelating structure that allows the central lanthanide to coordinate a labile water molecule.^2^ The most studied member of this series is **Gd** (hereafter, we will refer to the members of this series with the symbol of the lanthanide in bold), used as MRI contrast agent with the commercial name of Dotarem.^28^ The long rotational correlation time of this compound implies high proton relaxivities that can be otherwise achieved only using macromolecules.^29^**Dy** is also used in MRI as a contrast agent, sometimes with chemical modifications to the ligand structure.^30^ Some members of this series (particularly **Eu** and **Tb**)^31^ are often investigated for their high luminescence yields when chemically linked to chromophores.^32^ More recently, the magnetic properties of the late derivatives of the series have been investigated. **Dy** exhibits a giant field dependence of the relaxation time depending on the applied external magnetic field,^33^ capped square while the spin’s parity plays a crucial role in the appearance of slow relaxation of the magnetization at low temperatures.^21^ Nevertheless, the most intriguing property of these complexes is their magnetic anisotropy.^8,34^ Previous studies on the late lanthanides (**Tb** to **Yb**) showed that the easy direction (i.e., the axis most prone to be magnetized) varies by ca. 90° depending on the central ion.^20,21^ However, due to intrinsic sensitivity issues of standard single-crystal magnetometry, measurements could not be performed for **Ho** and **Tm**. The first half of the series, composed of ions with lower magnetic moments, remained to be investigated. Theoretical calculations demonstrated that, for **Dy**, the easy axis orientation is strongly dependent on the position of the hydrogen atoms belonging to the water molecule coordinated in apical position with relevant covalent effects.^35^

In this work, we characterize the magnetic anisotropy of all 13 magnetic derivatives of this series (from **Ce** to **Yb**) by combining cantilever torque magnetometry (CTM) and electron paramagnetic resonance (EPR) with *ab initio* calculations. We thus obtained a reliable and systematic library of the electronic structure and of the magnetic properties arising from the different occupations of the f orbitals of LnDOTA complexes. Our study compares isostructural light and heavy lanthanides, revealing an unnoticed trend: the *f*^*n*+7^ effect.

## Results and Discussion

**Figure 1:**
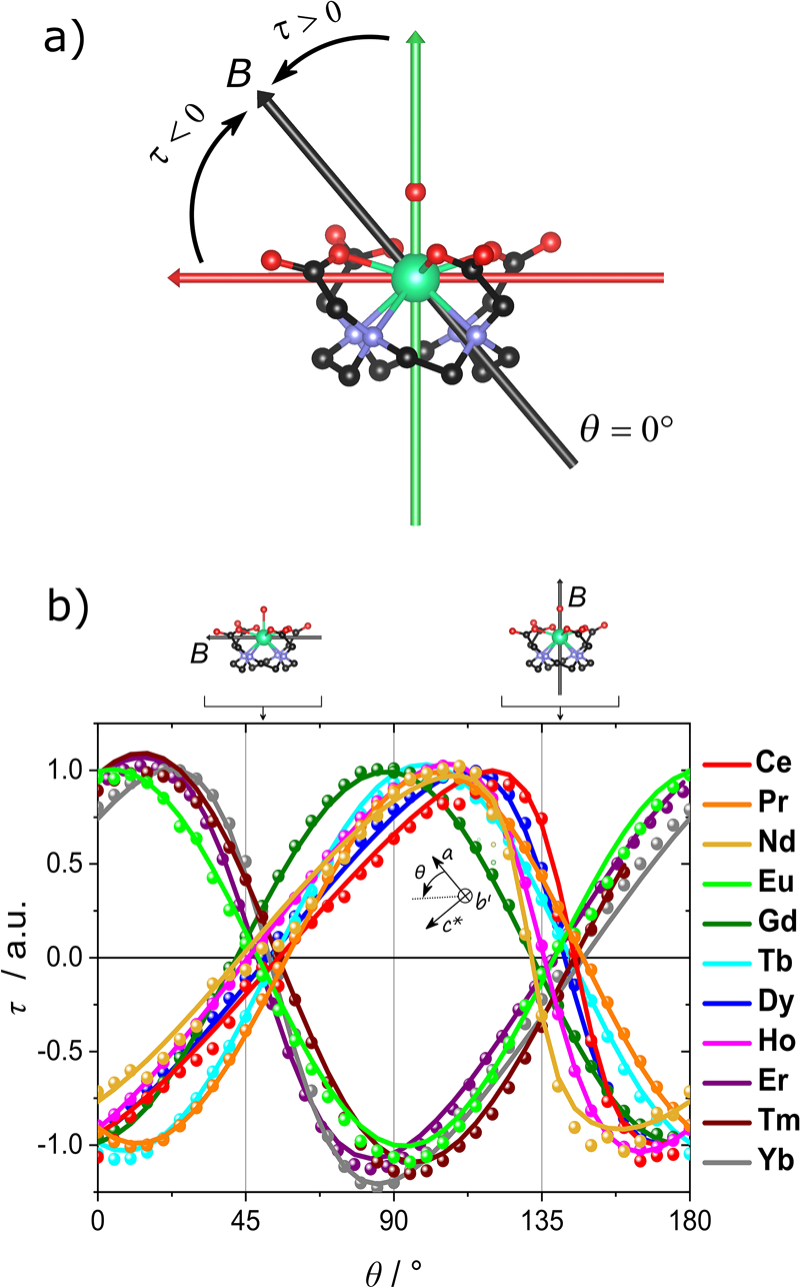
(a) Orientation of the magnetic field (black arrow) superimposed to the molecular structure at *θ* = 0°. The colored arrows represent the two observed different orientations of the easiest axis: ca. along the Ln–O_w_ bond and ca. perpendicular to the Ln–O_w_ bond (green and red arrow, respectively). Color code: green, Ln; black, C; red, O; blue, N. (b) Normalized experimental data (dots) and best fits (lines) obtained during Rot1 for all **Ln**. The temperature is 2 K for all derivatives except for **Er** (*T* = 5 K). The magnetic field is 12 T for **Ce** and **Nd**, 9 T for **Pr** and **Eu**, 5 T for **Er**, 3 T for **Gd**, **Tb**, and **Yb**, 2 T for **Dy** and **Ho**, and 1.5 T for **Tm**. On top of the graph we reported the orientation of the magnetic field (black arrow) with respect to the molecule at selected angles.

Magneto-structural correlations in the crystal phase can only be understood starting from the crystal structure of the investigated complex. The structures of several derivatives of this family of complexes have been reported in the literature,^36−38^ but we have redetermined all the structures (except the radioactive **Pm**) to give a consistent picture and to highlight subtle trends. These compounds crystallizes in the *P*1̅ triclinic space group. Once the cocrystallized water molecules are taken into account, the compounds can be described with the formula Na[Ln(DOTA)(H_2_O)]·4H_2_O. The synthetic procedure that we used to obtain crystals is described elsewhere.^33^ A detailed analysis of the structural changes along the series is reported in Table S1 and Figures S1–S5 and reveals that the DOTA^4–^ ligand acts, in the solid state, as a rigid scaffold that coordinates all the Ln ions in the same manner. A structure of the LnDOTA^–^ anionic complex is reported in Figure 1a. If one only considers the first coordination sphere, the closest geometry for all the complexes is capped square antiprismatic with the the *C*_4_ axis being along the Ln–O_w_ bond. However, it is now well established that this approximation cannot explain the magnetic anisotropy of these systems.^20,21,35^

Given the low symmetry of the crystal, only one magnetically inequivalent molecule is present in the unit cell, so the magnetic anisotropy tensors of the molecules can be unambiguously mapped using single-crystal measurements.^39^ While in previous works on heavy Ln complexes this type of measurement was achieved using standard single-crystal magnetometry,^20,21^ here we exploit the high sensitivity and simplicity of cantilever torque magnetometry.^40−44^ More details about the experimental setup and the basic principles of the technique can be found in the SI and in the literature.^45,46^ The magnetic torque (**τ**) is the vector product between magnetization (**M**) and magnetic field (**B**). In the low-field/high-temperature regime, it depends linearly on the magnetic anisotropy of the susceptibility tensor.^45^ For high fields and/or low temperature, the angular dependence of the torque becomes less trivial, but a fundamental characteristic is maintained: the torque vanishes when the field is parallel (easy zero) and perpendicular (hard zero) to the projection of the easiest magnetization direction (the lowest free energy direction) in the scanned crystallographic plane.

**Figure 2:**
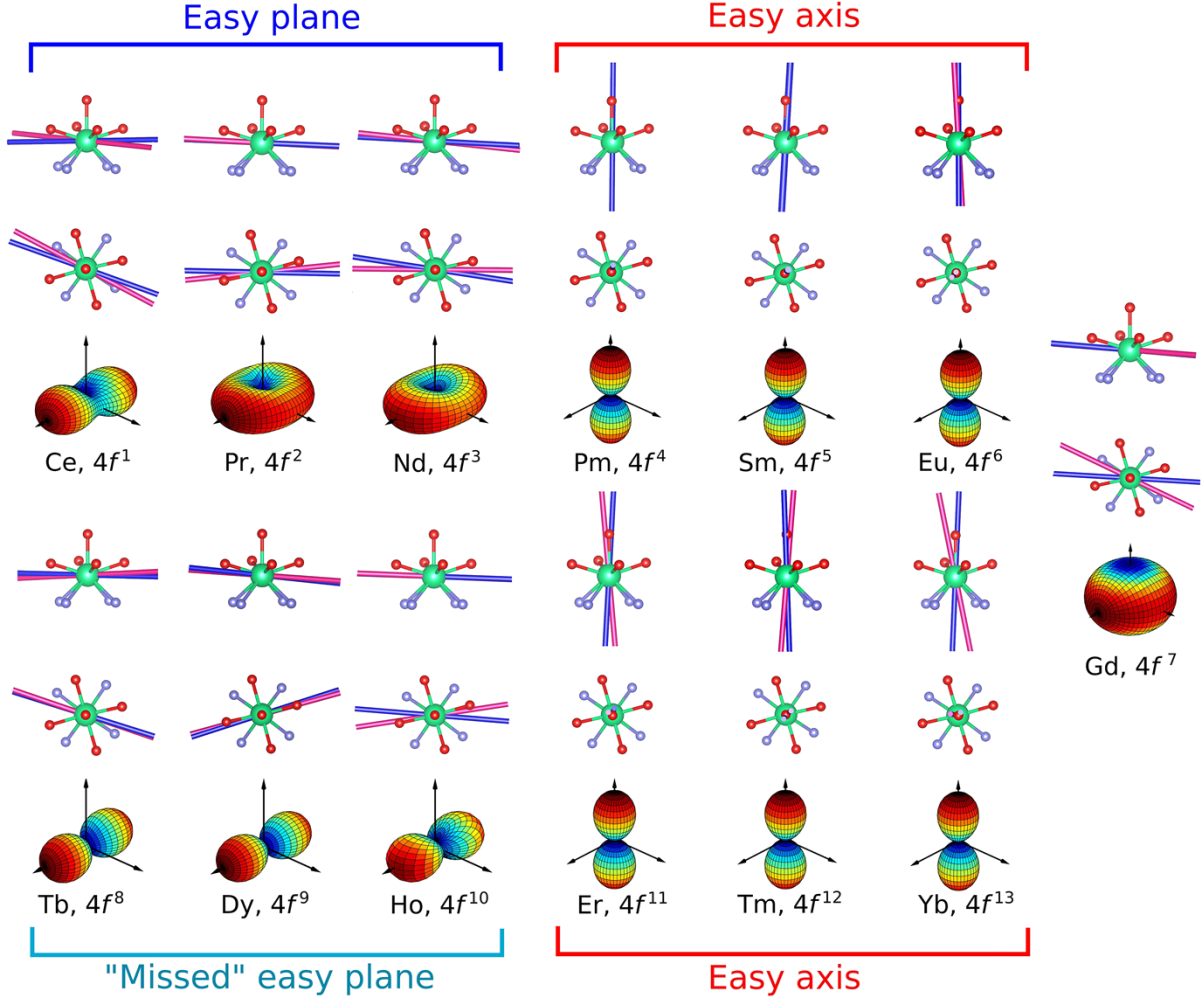
Experimental (pink) and theoretical (blue) orientation of the easiest axis of Ln. Data for **Tb** and **Yb** were taken from the literature. For **Sm** and **Pm**, only the theoretical calculations were performed. The colored 3D shapes are the *ab initio* calculated susceptibility tensor at *T* = 2 K and *B* = 1 nT. The vertical axis of the susceptibility plots corresponds to ca. the Ln-O_w_ bond. The ions are also classified according to the type of anisotropy they exhibit (see main text).

In Figure 1b, we report the angular dependence of the torque for all the investigated derivatives (Rot1, see SI for setup details). This rotation is particularly relevant because it allows sampling a crystallographic plane containing the lanthanide–water (Ln–O_w_) bond (deviation ca. 2°), i.e., the tetragonal pseudosymmetry axis. Considering the experimental setup described in the SI, at *θ* = 50° the magnetic field lies in the plane formed by the carboxylic oxygens of the ligand, while it is parallel to the Ln–O_w_ bond at ca. *θ* = 140°. The experimental curves can be grouped into two families, depending on the phase of the oscillation of the torque moment. Noticeably, the angular range in which the torque signal goes to zero is rather minute (40–60° and 130–150°), indicating that all the derivatives have the projection of the easiest direction either close to the Ln–O_w_ bond or almost perpendicular to it. In Figure 1a we have sketched the two orientations of the easy direction for the two groups of derivatives. For derivatives with a positive value of the torque at *θ* = 0°, the projection of the easiest direction in the ac* plane is close to the Ln–O_w_ bond (green arrow in Figure 1a). This is the case of **Eu**, **Er**, **Tm**, and **Yb**. On the contrary, for the easiest axis projection perpendicular to the Ln–O_w_ bond (red arrow in Figure 1a), the torque is negative at *θ* = 0°. This is the case of **Ce**, **Pr**, **Nd**, **Gd**, **Tb**, **Dy**, and **Ho**. To obtain an unambiguous determination of the orientation of the easiest axis, we performed a second rotation (Rot2) on those derivatives for which this direction was not previously experimentally determined (see Figure S6).^20,21^ The corresponding low-temperature data were fit with a phenomenological second-order spin Hamiltonian (see SI and Table S2 for details). The best fits of Rot1 are reported in Figure 1b (solid lines), while the fits of both Rot1 and Rot2 are reported in Figure S6. The experimentally determined easiest directions are reported in Figure 2 as pink axes; their director cosines are reported in Table S3. Although based on an oversimplified model, the fit of the experimental data allows extracting the low-temperature anisotropy of the susceptibility tensor (i.e., easy axis, easy plane, or rhombic) of the complexes. The results show that all but the first three derivatives exhibit an easy axis anisotropy. **Ce** and **Pr** were determined to be overall easy plane, with a non-negligible degree of rhombicity. **Nd** was also determined to be easy plane, but since the investigated crystal was very small, the rhombicity could not be detected.

**Figure 3:**
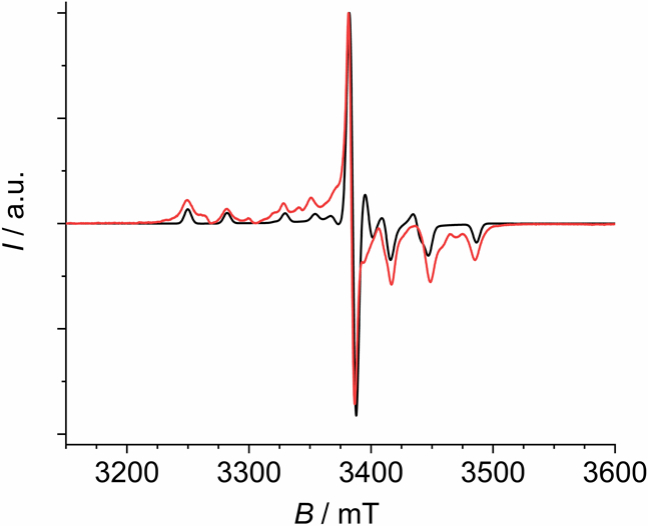
EPR W-band (frequency = 94.320 GHz) experimental spectrum (red trace) of **GdY** (doping level 6%) at *T* = 5.8 K and best simulation (black trace) obtained with the following Stevens parameters (in cm^–1^): *B*_2_^0^ = −7.5 × 10^–3^, *B*_4_^0^ = 8.5 × 10^–6^, *B*_2_^2^ = 2.5 × 10^–3^.

Kramers derivatives were also studied using cw-EPR. This technique has had historic importance in the understanding of the electronic structure of Ln-based systems,^47^ and its application to Ln-based molecular magnets gained impetus in the past decade.^10,16,48^ Only **Ce**, **Nd**, **Gd**, **Er**, and **Yb** showed a signal (Figures S7–S11). Their qualitative analysis agrees with the prediction of the anisotropy of the ground state by CTM: easy plane type spectra (i.e., *g*^eff^_*x,y*_ > *g*^eff^_*z*_) are observed for **Ce** and **Nd**, while easy axis type spectra (*g*^eff^_*z*_ > *g*^eff^_*x,y*_) are obtained for **Er** and **Yb**. Spectral simulations obtained using EASYSPIN^49^ provided the parameters reported in Table S4. **Gd** was more thoroughly investigated due to its potential use as a tag in protein structural determination via pulsed EPR.^50^ X-band (ca. 9.4 GHz) and W-band (ca. 94 GHz) EPR spectra at variable temperature (Figure 3 and Figure S11) allowed us to unambiguously determine for this derivative an easy axis type anisotropy, with non-negligible rhombicity in the hard plane (see SI and Figure S12 for a detailed discussion). The EPR-determined anisotropy agrees with the CTM results, but it is in contrast with the previous report of HF-EPR spectra at 240 GHz, where zero field splitting (ZFS) was deemed to be positive, albeit of absolute values close to those we obtain here. This discrepancy might originate from the low crystallinity of the previously investigated samples that were prepared by lyophilization.^51^ The latter process is indeed likely to remove water molecules from the sample, thus leading to a different structure and consequent difference in anisotropy.^35^

The static magnetic properties were characterized with standard magnetometry. The χ*T* curves are reported in Figure S13 and exhibit the typical decrease at low temperature due to crystal field (CF) splitting of the *J* multiplets. The magnetization curves for derivatives not previously measured are reported in Figure S14.

Concerning the relaxation dynamics (reported in Figures S15–S24), the parity trend observed for the second half of the series is here extended to the complete series: only Kramers ions show in-field Single Molecule Magnet (SMM) behavior. A comparison of dc–ac data easily shows that for all the measured derivatives the relaxation pathway is unique (Figures S25–S29) except for **Gd** (Figures S21 and S22), which shows a double peak, as previously reported for GdEDTA.^52^ The dilution in the diamagnetic analogue restores a single relaxation pathway also in **Gd** (Figures S23, S24, and S29). The relaxation times extracted from the fits are reported in Figure S30.

To check whether theory could reproduce the experimental torque results and to complete the series, we decided to employ state-of-the-art *ab initio* calculations, considering both ionic and covalent contributions at the highest affordable level of theory (see Computational Details in the SI and Table S5). The chosen molecular model included the lanthanide ion, the DOTA ligand, and the apical water molecule directly bonded to the lanthanide, in accordance with the M2m model proposed by Briganti *et al*.^35^ The similar cell parameters throughout the series and the isostructural nature of the crystal packing justify the employment of the same model for all derivatives. The main values of the susceptibility tensor at low temperature, *g* values, and CF parameters extracted from the calculation are reported in Tables S6–S9. The energy level structure and composition of the low-lying states are reported in Tables S10 and S11.

With theory and experiments at hand, we have simulated the torque curves for all the derivatives (Figures S31–S40). The low-temperature anisotropy is correctly predicted for all derivatives except for the quasi-isotropic **Gd**. The *ab initio* easiest directions are reported in Figure 2 as blue axes. Moreover, a 3D plot of the computed susceptibility tensor at low temperature is reported for each derivative in Figure 2. A comparison with the EPR results (compare Table S4 and Table S7) highlights a previously noticed tendency to overestimate axiality (e.g., in **Ce** and **Er**).^39^ The predicted orientation of the easiest axis matches very well the experimental one for all derivatives (minimum deviation: 3°, maximum deviation: 14°, average deviation over the 10 anisotropic derivatives experimentally investigated: 9°), as reported in Table S3. Importantly, the easy axis of **Ho** was here calculated to be at almost 90° from the Ln–O_w_ bond, in agreement with experiments but in contrast with previous predictions.^21^ This highlights the importance of the choice of the appropriate theoretical model, which must accurately reproduce the molecule beyond the first coordination sphere (here orientation of the H atoms of the water molecule induced by the next neighbors).

The experimental χ*T* curves (Figure S13) are well reproduced by the calculation only for **Pr**, **Nd**, **Gd**, **Tb**, and **Dy**. The discrepancies can be justified for **Eu** and **Sm** (which possess a poorly magnetic ground state), but their explanation is challenging for the other derivatives.

The investigation of the full series allows an unprecedented birds-eye view on the magnetic anisotropy of isostructural series. By comparing the orientation of the easiest axis, we noticed that its orientation is shared by Ln^3+^ having an external configuration differing by seven f electrons (see Table 1). The experimental directions for the investigated couples are all very similar, especially considering the experimental error of 5–10°. The *ab initio* results are strikingly similar for all the couples except for **Pr**/**Dy**. We attribute this discrepancy to the extremely low and quasi-easy plane anisotropy of **Pr**, which renders the identification of the easiest direction in the easy plane rather difficult.

**Table 1:** Angle between the Easiest Direction of Derivatives Differing by Seven f Electrons

derivatives	exptl angle/deg	*ab initio* angle/deg
**Ce** (4f^1^)/**Tb** (4f^8^)	12	2
**Pr** (4f^2^)/**Dy** (4f^9^)	11	21
**Nd** (4f^3^)/**Ho** (4f^10^)	10	4
**Pm** (4f^4^)/**Er** (4f^11^)		7
**Sm** (4f^5^)/**Tm** (4f^12^)		2
**Eu** (4f^6^)/**Yb** (4f^13^)	12	1

A closer inspection of the theoretical calculations (Table S12) reveals that also the other axes (intermediate and hard) coincide for **Ce**/**Tb**, **Nd**/**Ho**, and **Pm**/**Er** but deviate significantly for **Sm**/**Tm** and **Eu**/**Yb**. This can be understood following the same reasoning adopted for **Pr**: the anisotropy of **Sm** and **Eu** is small but pronouncedly axial. Therefore, the principal directions in the hard plane are challenging to identify. This problem is also relevant in the experiments: among the derivatives for which we have performed two rotations, only one showed a sufficiently rhombic anisotropy to identify the complete reference frame: **Ce**. In this way, we obtained the experimental confirmation that **Ce** and **Tb** share the same anisotropy reference frame (5°, 11°, and 12° deviation between hard, intermediate, and easy axes, respectively). We have graphically reported the experimental and theoretical orientation of the main axes for the couple **Ce**/**Tb** superimposed on the same structure in Figure 3a and b, respectively.

A rather intuitive explanation of the *f*^*n*+7^ effect could be phrased as follows: adding seven electrons in seven f orbitals of isostructural lanthanide complexes is equivalent to adding a sphere of negative charges, which should not affect the magnetic anisotropy.^53^ This simple explanation suggests that both orientation and type of anisotropy should be similar for pairs of ions differing by seven f electrons. However, only the former is verified here (compare the susceptibility tensors reported in Figure 2).

**Figure 4:**
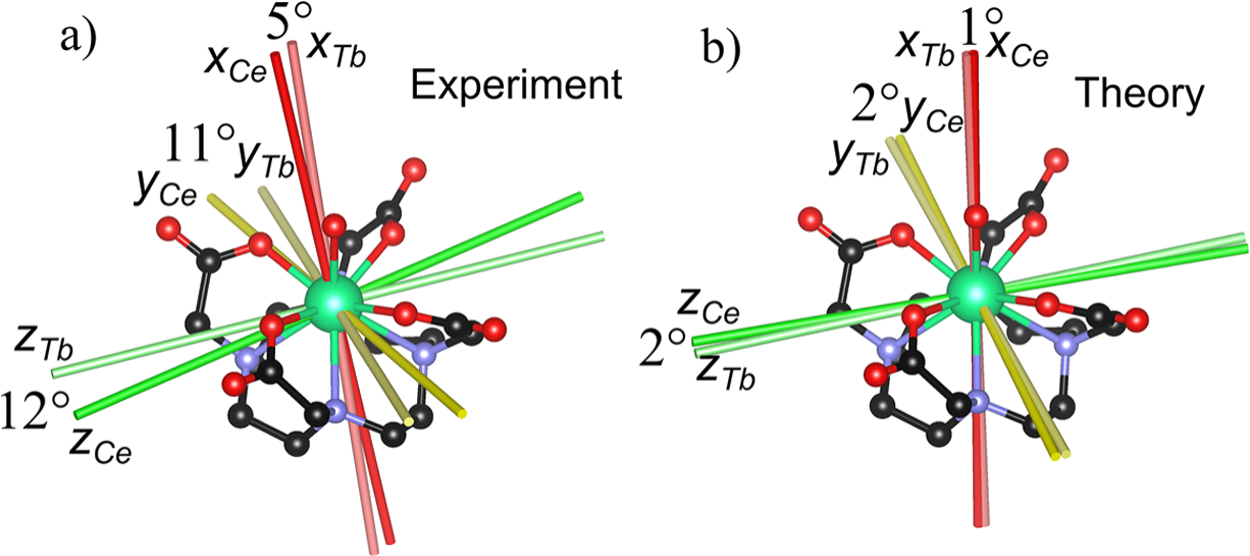
(a) Experimental and (b) *ab initio* magnetic reference frame of **Ce** and **Tb** superimposed on the same structure.

**Figure 5:**
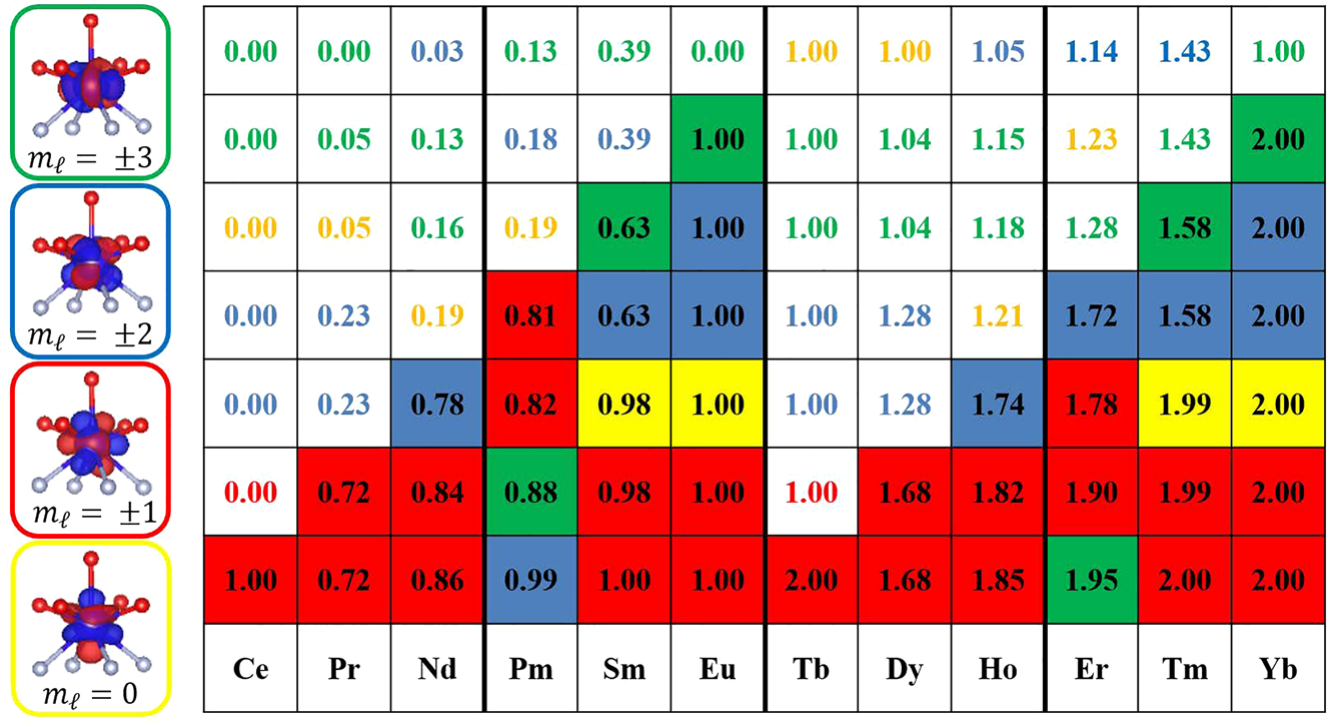
*Ab initio* predicted 4f orbital occupancy for the ground state for all the anisotropic derivatives of the series. The color code follows the legend on the left side of the picture, where the f orbitals have been superimposed to a simplified molecular structure. Fully colored cells indicate the most representative (population >0.5) spin-up (first half) or spin-down (second half) configuration of the 4f electrons. The thick black lines emphasize the turning points of the orientation of the easy magnetic axis along the series.

*Ab initio* calculations reveal that the CASSCF-RASSI 4f orbitals associated with the different *m*_*l*_ values, assuming the pseudo-*C*_4_ axis of the complex as the quantization axis (Figure S41), have the relative occupation shown in Figure 5. First, we notice a striking similarity between pairs of ions differing by seven f electrons. The minor differences observed for the couple **Pm**/**Er** can be justified by recalling the absence of an experimentally determined structure for **Pm**. We also point out that the two *m*_*l*_ = ±1 orbitals have a high occupancy for all the derivatives. This agrees with the Aufbau principle in the presence of a CF, given the substantially nonbonding character (and thus lower energy) of these orbitals (see the pictorial view of the orbitals reported in Figure 5). The occupation of the other orbitals is instead dictated by the competition of the Aufbau principle and Hund’s rules. Emblematic is the case of **Eu** and **Tm**, for which the occupation of the orbitals with *m*_*l*_ = ±2, ±3 provides for the two components a 0.6e^–^/0.4e^–^ ratio, supporting the establishment of concomitant competitive Aufbau and Hund’s regimes (see Figure 4). The result is an orbital filling in the order *m*_*l*_ = |2| < |3| < 0 < |2| < |3| from electron 3(+7) to electron 6(+7). The analysis of the f orbitals’ occupation trend could also be used to qualitatively predict the change of the orientation of the magnetic anisotropy. We notice that the turning points of the orientation of the easy axis (**Pm** and **Er**, see Figure 2) happen when Hund’s rules start to dominate over the Aufbau regime, resulting in the population of the *m*_*l*_ = |3| rather than of the second *m*_*l*_ = |2|. Such analysis has been possible only thanks to the availability of the whole series. The analysis of the f orbitals’ occupation trend finely explains the *f*^*n*+7^ effect and becomes a candidate to be considered as a reliable qualitative tool for the prediction of the magnetic anisotropy orientation. Finally, such results indicate that the occupation of the f orbitals depends on a subtle balance among CF, spin–orbit coupling, and electron–electron interactions and, therefore, can change depending on the nature, number, and geometry of the ligators around the lanthanide ion.

In a qualitative picture, the *ab initio*-computed magnetic anisotropy of **Pm**-**Eu**/**Er**-**Yb** confirms the *f*^*n*+7^ correspondence for both orientation and nature of the magnetic anisotropy. On the contrary, we computed (slightly rhombic) easy plane anisotropy of the ground state of **Ce**, **Pr**, and **Nd**, while **Tb**, **Dy**, and **Ho** provide strong easy axis anisotropy, as experimentally observed. This can be rationalized by considering the *m*_*j*_ composition of the states (*m*_*j*_ being the projection of the total angular momentum along *z*), when the quantization axis is fixed along the easiest direction (that is, the usual convention). Indeed, for **Tb**, **Dy**, and **Ho** we retrieve a composition dominated by high *m*_*j*_ values (Tables S10 and S11). Noticeably, for ions with large *J* values, the axiality can only be quenched at a high level of perturbation.^53,54^ This is the reason why Dy^3+^ complexes are often strongly axial even in “unfavorable” equatorial CF environments, such as the one provided by DOTA.^53,54^

Interestingly, the *f*^*n*+7^ correspondence is fully restored (orientation *and* shape) if we calculate the susceptibility tensor at 100 K (see Table S15 and Figure S42). In this regard, a close look at the computed energy level structure (Figure S43) is telltale. While the first three derivatives have a well-isolated ground state, **Tb**, **Dy**, and **Ho** exhibit several low-lying states. These energy levels contribute to the magnetization along different directions, as evident from the composition of the states expressed fixing the quantization axis along the pseudo-tetragonal axis (see Tables S13 and S14). Such a result can be interpreted as a “missed” easy plane magnetic anisotropy in the second half of the series due to low-symmetry components of the CF.

Finally, our *ab initio* analysis allows appreciation of the strong correlation between the asphericity of the 4f electron density^17,19^ and the magnetic anisotropy, recently experimentally investigated through high-resolution synchrotron X-ray diffraction.^55^ Especially for **Tb** and **Dy**, a deviation from the 4-fold symmetry is evident in the calculated electron density around the metal ion (see Figure S44). When observed from the pseudo-tetragonal axis, we can recognize a compressed shape, with the lowest electron density being coincident with the easiest magnetization axis.

## Conclusion

A correlation in the easiest direction between ions differing by seven f electrons is here experimentally found and theoretically predicted in both qualitative and quantitative ways. Qualitatively, an approach based on the orbital ladder occupation has been presented, while quantitatively state-of-the-art *ab initio* calculations provided sets of CF parameters that were used to accurately reproduce and rationalize the single-crystal experiments. The magnetic anisotropy orientation significantly changes along the series: **Ce**, **Pr**, and **Nd** have (almost) easy plane anisotropy tensors, while the other derivatives (except **Gd**) are strongly easy axis. **Tb**, **Dy**, and **Ho** show an easy axis anisotropy that can be identified as a “missed” easy plane anisotropy. Interestingly, the similarity becomes again evident at high temperature, stressing the importance of defining the external conditions (temperature and magnetic field) when discussing magnetic anisotropy.^8,34^

Although the *f*^*n*+7^ trend might seem trivial for highly symmetric systems with essentially electrostatic bonds, it is not so for *C*_1_ symmetric molecules, such as LnDOTA, where asymmetries in the CF and covalency play a crucial role.^35^ The fact that this effect was observed in the LnDOTA complexes is a promising sign that the effect could be extended to other isostructural series of poorly symmetric molecules. Remarkably, low-symmetry molecules constitute more than 90% of the lanthanide complexes reported to date in the Crystallographic Cambridge Database.

Moreover, this trend could provide valuable shortcuts to save huge amounts of computational power. Indeed, it will also constitute a rather convenient way to gain information on lanthanides that have a relatively small total angular momentum and are thus difficult to investigate experimentally. It would also be interesting to test the limits of the *f*^*n*+7^ effect in terms of covalency of the metal–ligand interaction.

Finally, the calculation of electronic structure of the whole LnDOTA series made possible shedding further light on the non-negligible role of the CF in lanthanide complexes. Indeed, we showed here the possibility to exploit the Aufbau vs Hund’s rule competition to finely tune the magnetic anisotropy of lanthanides. This plays the same role as the hard or soft nature of the coordinating atoms in determining the high spin–low spin configuration in transition metal complexes.

## Supporting Information Available

Crystallographic characterization; torque measurements and analysis; EPR spectra and analysis; magnetic measurements and simulations; energy level structure and composition; orbital occupancy (PDF)

## Accession Codes

CCDC 2032925–2032926, 2032928–2032929, 2032931, 2032934–2032938, 2032946, and 2070879 contain the supplementary crystallographic data for this paper. These data can be obtained free of charge via www.ccdc.cam.ac.uk/data_request/cif, or by emailing data_request@ccdc.cam.ac.uk, or by contacting The Cambridge Crystallographic Data Centre, 12 Union Road, Cambridge CB2 1EZ, UK; fax: +44 1223 336033.
