# Supplementary material for: Magnetic Anisotropy Trends along a Full 4f-Series: The fn+7 Effect
Source: J Am Chem Soc. 2021 May 24;143(21):8108–15. doi: 10.1021/jacs.1c02502 (PMC8297734; doi:10.1021/jacs.1c02502)
Supplement: Supplementary file 1 — ja1c02502_si_001.pdf [file ja1c02502_si_001.pdf]

SUPPORTING INFORMATION

for

Magnetic anisotropy trends along a full 4f-series: the f^{n+7} effect

Matteo Briganti[a], Eva Lucaccini[a], Laura Chelazzi[a,b], Samuele Ciattini[a,b], Lorenzo Sorace[a], Roberta Sessoli[a], Federico Totti[a], and Mauro Perfetti[a]

[a] Department of Chemistry ‘‘U. Schiff’’, University of Florence, Via della Lastruccia 3-13, 50019, Sesto Fiorentino (FI), Italy.

[b] Center of Crystallography, University of Florence, Via della Lastruccia 3, 50019 Sesto Fiorentino (FI), Italy.

Index

Crystal structure 3

Experimental determination of the easiest direction 6

Director cosines of the easy axis 8

EPR 9

Dc measurements 14

Ac measurements 16

- Cerium 16
- Neodymium 17
- Samarium 18
- Gadolinium 19
- Yttrium-Gadolinium 20
- Susceptibility - dc vs ac measurements 21
- Relaxation times 23

Ab initio model 24

Ab initio results 25

CTM simulations using the *ab initio* results 30

Other tables and Figures 43

References: 49

Crystal structure

All derivatives of the series were synthesized as previously described in literature.[1] The compounds were obtained in form of large single crystals suitable for X-ray diffraction and torque measurements. The structures were collected at ca. 100 K on either an Oxford Diffraction X'Calibur diffractometer (Cu Kα (l = 1.54056 Å) radiation) equipped with a CCD detector or a Bruker Apex II CCD diffractometer (Mo Kα (l = 0.7107 Å) radiation). Absorption correction was performed with the SADABS software. SIR2014 was used for structure solution and refinement based on F^2. Non-hydrogen atoms were refined anisotropically. The hydrogen atoms of the apical water molecule were added to the structures only when experimentally observed.

Ln	a / Å	b / Å	c / Å	α / °	β / °	γ / °	V / Å^3
Ce	8.6228(4)	9.2425(6)	15.8103(7)	82.879(5)	84.521(4)	80.459(5)	1229.39
Pr	8.6344(17)	9.2352(17)	15.793(3)	82.936(7)	84.627(7)	80.616(7)	1229.57
Nd	8.6217(4)	9.1798(4)	15.7319(7)	82.898(4)	84.797(4)	80.846(4)	1216.61
Sm	8.6202(13)	9.1620(12)	15.653(2)	82.866(5)	84.951(5)	80.983(5)	1209.01
Eu	8.6385(12)	9.1550(12)	15.658(2)	83.005(5)	85.075(5)	81.242(5)	1211.91
Gd	8.6325(10)	9.1434(10)	15.6275(17)	82.696(4)	85.179(4)	81.276(4)	1207.35
Tb	8.6396(17)	9.1325(17)	15.606(3)	83.050(8)	85.334(8)	81.465(8)	1206.22
Dy	8.6514(18)	9.0997(16)	15.551(13)	82.995(7)	83.358(8)	81.507(7)	1199.32
Ho	8.6458(12)	9.1005(12)	15.548(2)	83.079(2)	85.561(5)	81.628(5)	1199.25
Er	8.6556(16)	9.0939(17)	15.516(3)	83.062(6)	85.549(5)	81.573(5)	1197.05
Tm	8.6591(13)	9.0838(11)	15.502(2)	83.136(5)	85.726(6)	81.707(5)	1195.91
Yb	8.6555(15)	9.0736(15)	15.465(3)	83.126(6)	85.785(6)	81.759(6)	1191.42

Table S1 Cell parameters of all the investigated derivatives.

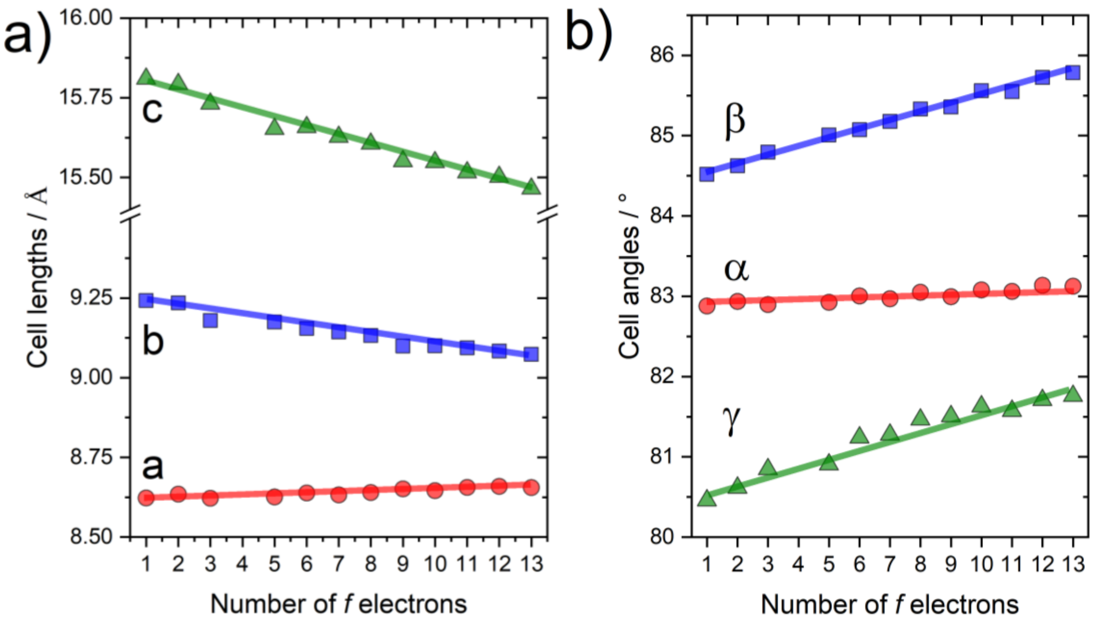

Figure S1. Graphical view of the evolution of the cell parameters (a) lengths and b) angles) along the series.

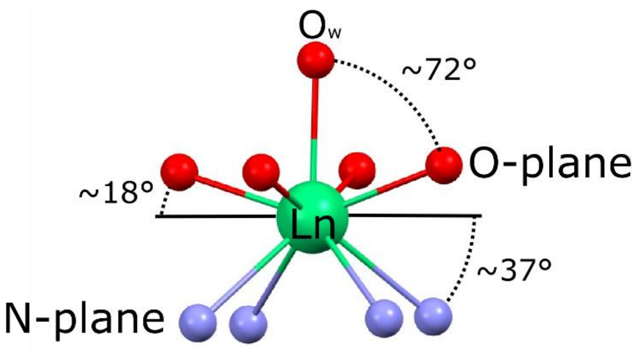

Figure S2. Description of the first coordination sphere around the Ln ion. The reported angles are averaged on the four oxygen (or nitrogen) atoms and along the entire series.

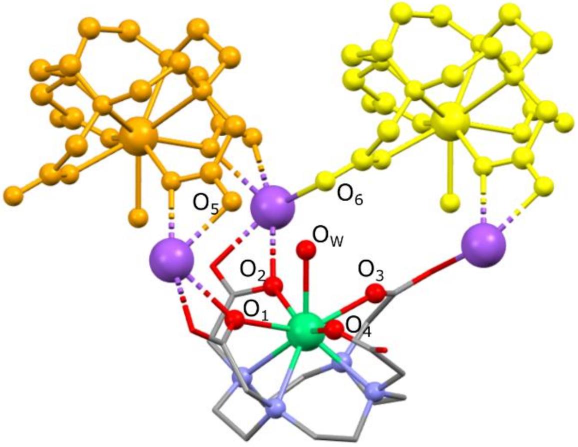

Figure S3. View of the packing. The labels of the oxygen atoms correspond to the labels reported in Figure S4. O_5 and O_6 are the closest oxygen atoms belonging to neighboring molecules (orange and yellow). Color code: Ln: green, O: red, N: blue, C: gray, Na: violet.

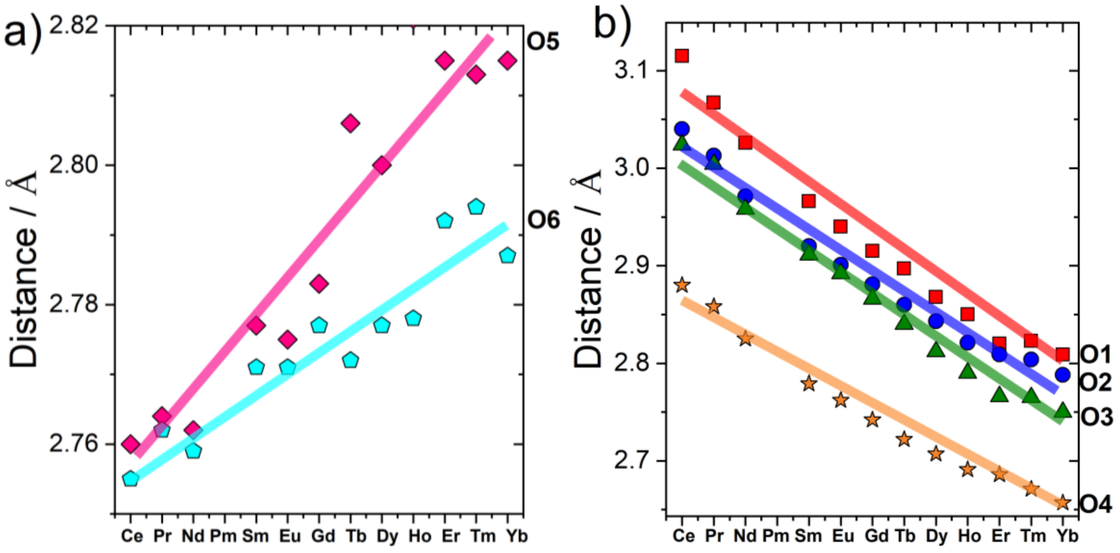

Figure S4. Distances between the oxygen of the apical water molecule (O_W) and a) the two closest carboxylic oxygens of the neighboring molecules and b) the four coordinated carboxylic oxygens. The lines are a guide to the eye.

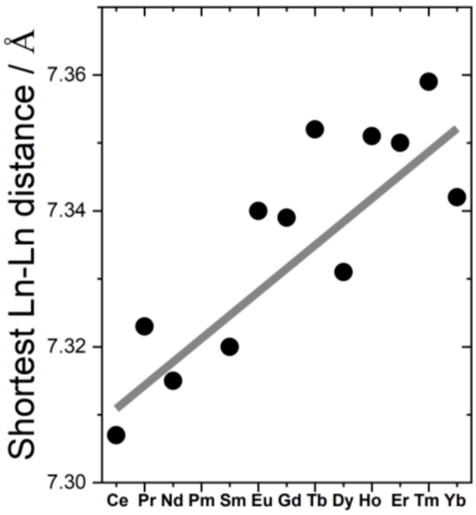

Figure S5. Shortest Ln-Ln distance in the crystal lattice. The line is a guide to the eye.

Experimental determination of the easiest direction

We performed the measurements using a home made two-legged CuBe cantilever separated by 0.1 mm from a gold plate. The cantilever was inserted into an Oxford Instruments MAGLAB2000 platform with automated rotation in a vertical magnet. The capacitance was detected with an Andeen-Hagerling 2500A Ultra Precision Capacitance Bridge.

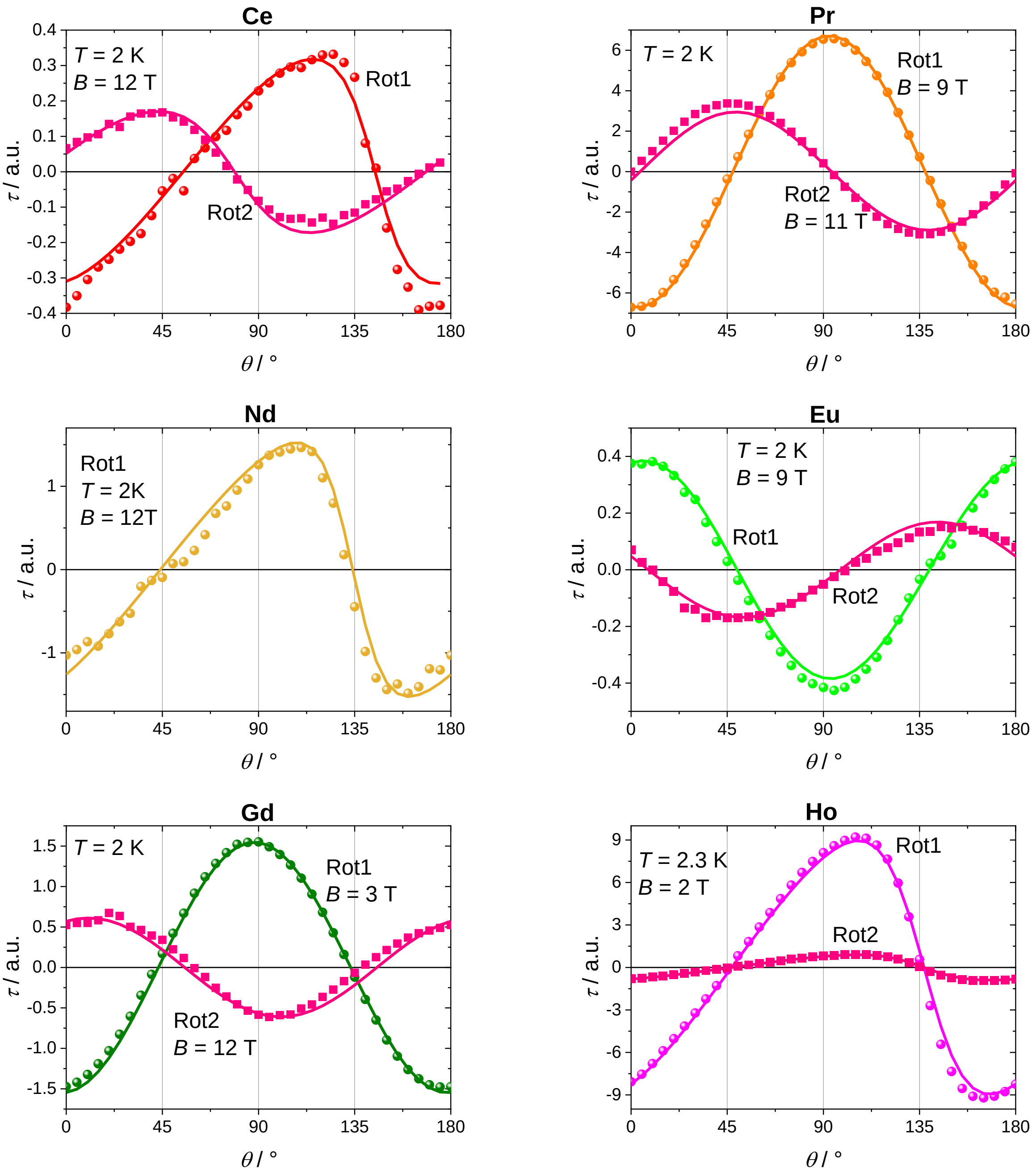

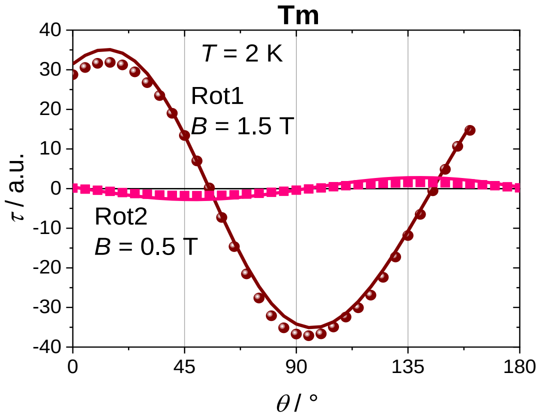

Figure S6. Fits (with fictitious b_2^0 and b_2^2 parameters) of the experimental torque for all the derivatives with previously unknown direction of the easy axis. The fit for **Eu** was performed using a $J = 1$ ground state. Rot2 for **Nd** did not give a detectable signal. The magnetic field (***B***) at the beginning of Rot1 and Rot2 was along (-0.989,0.150,0) and (0.150,0.989,0), respectively. The rotation axis (***Y***) for Rot1 and Rot2 was (-0.150,-0.989,0) and (-0.989,0.150,0), respectively. The only exceptions are: Rot2 for **Ce** (***B*** = (0,-1,0) and ***Y*** = (-0.643, 0, -0.766)), and Rot2 for **Gd** (***B*** = (0.150,0.989,0) and ***Y*** = (0,0,-1)).

The Hamiltonian used for the fits in Figure S6 was composed by a crystal field and a Zeeman term:

$$\mathcal{H} = b_2^0\hat{\boldsymbol{O}}_2^0 + b_2^2\hat{\boldsymbol{O}}_2^2 + g_J\mu_B\hat{J}\cdot\boldsymbol{B}$$

Where b_2^0 ($\boldsymbol{O}_2^0$) and b_2^2 ($\boldsymbol{O}_2^2$) are the 2nd order axial and rhombic parameter (operator), g_J is the appropriate Landè g-factor, μ_B is the Bohr magneton, $\hat{J}$ is the total angular momentum operator and **B** is the magnetic field.

Derivative	Sign of b_2^0	b_2^2/b_2^0
**Ce**	+	1
**Pr**	+	0.79
**Nd**	+	n.d.
**Eu**	-	0
**Gd**	-	0.62
**Ho**	-	0.2
**Tm**	-	0

Table S2. Sign of the axial CF b_2^0 (positive: easy plane, negative: easy axis) and degree of rhombicity b_2^2/b_2^0 (1 maximum rhombicity, 0 axial). The absolute values of the parameters are not reported because the mass of the crystals is known with poor precision. N.d.=not detectable.

Director cosines of the easy axis

Ln	z-axis, exp.			z-axis, calc.			ζ / °	ε / °	
	a	*b'*	*c**	*a*	*b'*	*c**		calc.	exp.
**Ce**	0.569	0.131	0.812	0.640	0.335	0.692	14	89	84
**Pr**	0.674	-0.107	0.731	0.671	0.017	0.741	7	88	87
**Nd**	0.719	0.001	0.695	0.670	0.148	0.727	9	88	84
**Pm**	-	-	-	-0.748	-0.017	0.664	-	6	-
**Sm**	-	-	-	-0.728	0.030	0.687	-	6	-
**Eu**	-0.760	0.093	0.643	-0.727	0.005	0.684	5	4	2
**Gd**	0.635	0.428	0.643	0.706	0.052	0.707	22	85	88
**Tb**	0.572	0.328	0.752	0.635	0.278	0.720	4	90	86
**Dy**	0.671	-0.286	0.683	0.700	-0.015	0.717	3	82	85
**Ho**	0.665	-0.153	0.731	0.668	0.079	0.739	13	88	87
**Er**	-0.819	0.016	0.574	-0.724	-0.137	0.676	12	13	7
**Tm**	-0.807	0.142	0.574	-0.743	0.018	0.669	10	4	6
**Yb**	-0.877	0.040	0.479	-0.739	0.016	0.674	14	5	12

Table S3. Director cosines of the lowest free energy direction (easiest axis) in the orthogonal *ab'c** reference frame for all the investigated derivatives obtained by experiments and calculations. The angle ζ represents the fitted-calculated agreement. The angle ε is the angle between the calculated or experimental axis and the Ln-O_w bond (dir. cosines: (-0.761, 0.080, 0.643) in *ab'c**).

EPR

X-band EPR spectra (ν ≈ 9.5 GHz, exact frequency reported in the caption of each spectrum) were recorded on a Bruker Elexsys E500 spectrometer equipped with an ESR900 (Oxford Instruments) continuous-flow ^{4}He cryostat to work at low temperature and a SHQ resonator. The crystalline powder of each sample was ground and pressed in pellet to avoid preferential orientation and then placed in 4 mm diameter quartz tubes. EPR spectra (ν ≈ 94 GHz) of **Gd** were recorded on a Bruker E600 continuous-wave spectrometer with cylindrical cavity equipped with a split-coil superconducting magnet that generates a horizontal magnetic field (Oxford Instruments). Temperature was controlled with a continuous-flow cryostat (Oxford CF935), operating from room temperature down to 4.2 K. Ground powder was dispersed in wax to avoid preferential orientation of the micro-crystallites due to magnetic torque, and inserted in 0.8 mm diameter quartz tube to perform the measurement. Notwithstanding this procedure, spectra clearly shows the additional contribution of a single individual crystal which is not powder averaged.

Simulations of the derivatives containing anisotropic Kramers' ions providing an EPR signal (**Ce, Nd, Er, Yb**) were performed using EasySpin[2] on the basis of an effective doublet spin Hamiltonian, including hyperfine coupling where needed (i.e. except for **Ce**):

$$\widehat{H}_{EPR} = \mu_B \widehat{\boldsymbol{S}}^{\boldsymbol{eff}} \cdot \boldsymbol{g}^{\boldsymbol{eff}} \cdot \vec{\boldsymbol{B}} + \widehat{\boldsymbol{S}}^{\boldsymbol{eff}} \cdot \boldsymbol{A}^{\boldsymbol{eff}} \cdot \hat{\boldsymbol{I}}$$

For **Gd**, a Spin Hamiltonian acting on the *S* = 7/2 ground multiplet was considered, with isotropic *g* value (*g* = 1.995), rhombic second order contributions and only axial fourth order terms (Stevens' notation)[3]:

$$\widehat{H}_{Gd} = \mu_B g \widehat{\boldsymbol{S}} \cdot \vec{\boldsymbol{B}} + \mathrm{B}_2^0 \widehat{\boldsymbol{O}_2^0} + B_2^2 \widehat{\boldsymbol{O}_2^2} + B_4^0 \widehat{\boldsymbol{O}_4^0}$$

Best simulation parameters are reported in Table S4, and comparison between experimental and simulated spectra are reported in Figure S7-S11. For the sake of clarity, in Figure S12 we report simulation of **Gd** obtained by assuming a reverse sign for the Spin Hamiltonian parameters with respect to the best fit ones reported on main text. While this set still provides the correct resonance fields, it clearly fails to correctly reproduce the temperature dependence of the spectra. In all cases, pseudo-Voigtian lines were assumed, with isotropic H-Strain superimposed on them to account for unresolved hyperfine coupling.

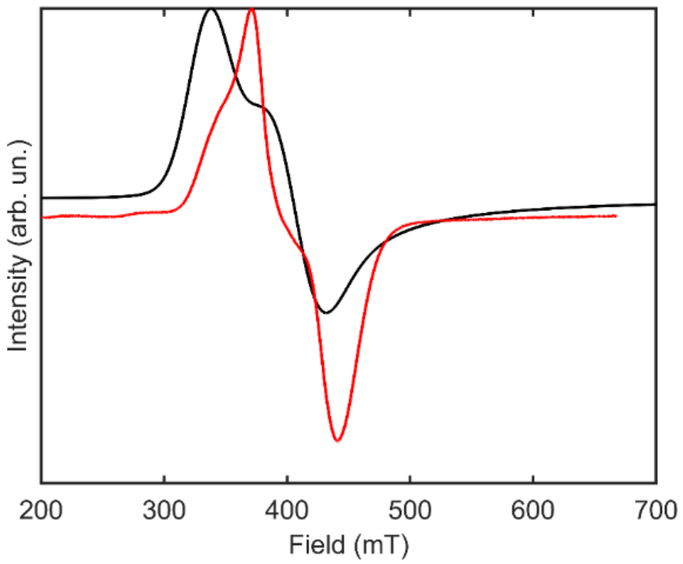

Figure S7. EPR X-band (ν = 9.4308 GHz) experimental spectrum (red trace) of **Ce** measured at 5 K and best simulation (black trace) obtained with parameters reported in Table S4.

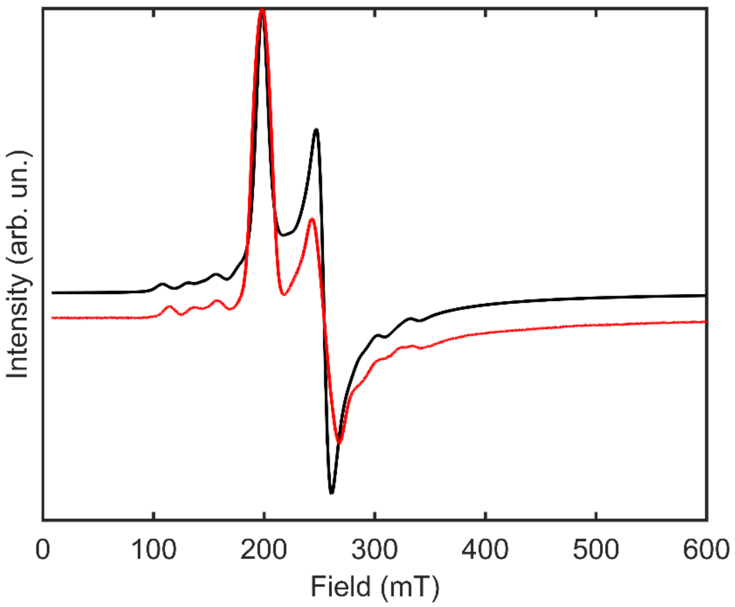

Figure S8. EPR X-band (ν = 9.4270 GHz) spectrum of **Nd** measured at 5 K and best simulation obtained with parameters reported in Table S4.

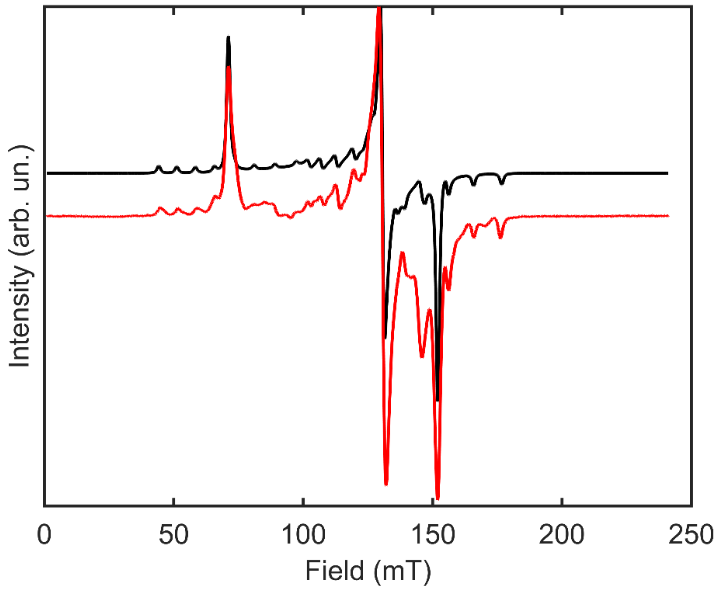

Figure S9. EPR X-band (v = 9.6398 GHz) spectrum of **YEr** (doping level 5%) measured at 5 K and best simulation obtained with parameters reported in Table S4.

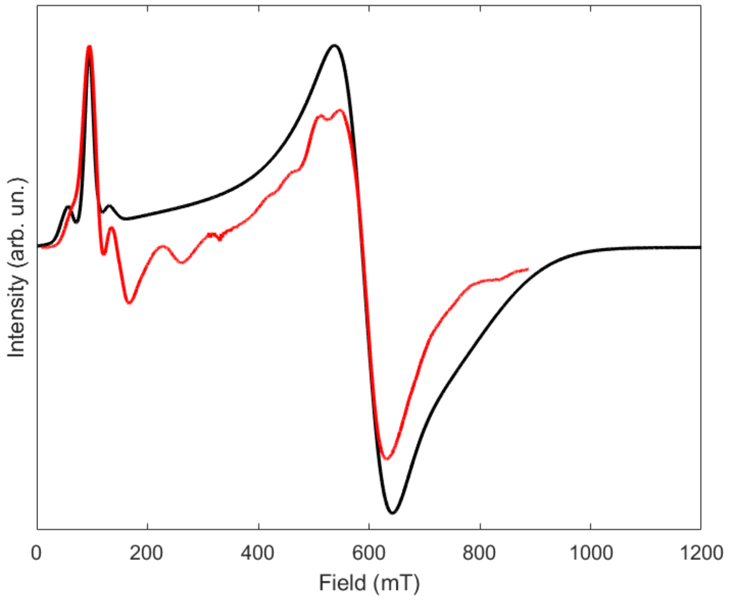

Figure S10. EPR X-band (v = 9.4046 GHz) spectrum of **Yb** measured at 5 K and best simulation obtained with parameters reported in Table S4.

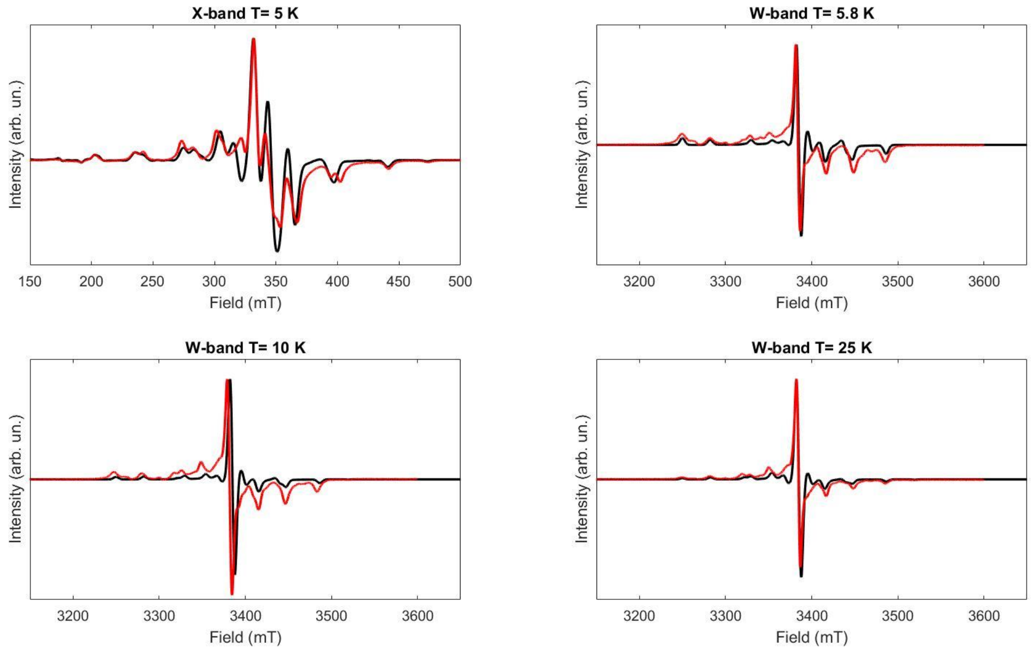

Figure S11. EPR X- (freq = 9.4200 GHz) and W-band (freq = 94.320 GHz) experimental spectra (red traces) of **GdY** (doping level 6 %) at different temperatures and best simulation (black traces).

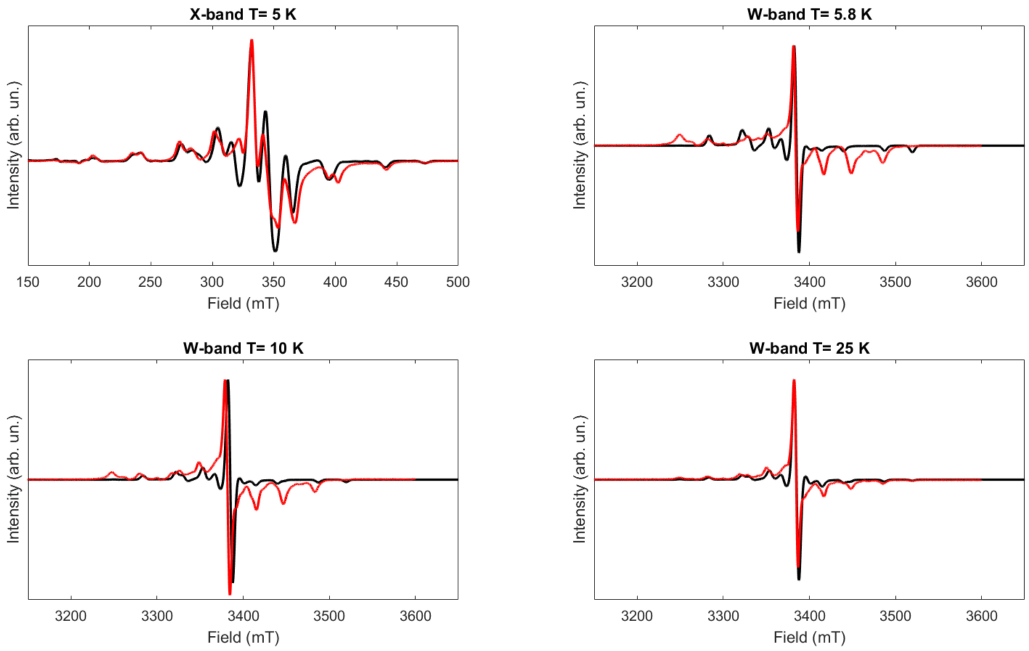

Figure S12. Comparison between experimental (red traces) and simulated (black traces) EPR spectra assuming opposite signs of the Spin Hamiltonian parameters with respect to those reported in Table S4 and Figure 3.

	$g_{eff.x}$	$g_{eff.y}$	$g_{eff.z}$	$A_{eff.x}$/ MHz	$A_{eff.y}$/ MHz	$A_{eff.z}$/ MHz
**Ce**	3.00	1.75	1.15	-	-	-
**Nd**	3.40	2.65	<0.6	1200	900	n.d.
**Er**	4.53	5.27	9.68	480	554	1026
**Yb**	0.90	1.12	7.022	n.d.	n.d.	1900
	B_2^0 /cm^{-1}	$\lvert B_2^2 \rvert$/cm^{-1}	B_4^0/cm^{-1}			
**Gd**	-7.5(4) 10^{-3}	2.5(1) 10^{-3}	8.5(2) 10^{-6}	-	-	-

Table S4. Best simulation parameters of the EPR spectra reported in Figure 3 (main text) and Figures S7-S11. Values of A^{eff} refer to the most abundant nuclear spin active isotope. (n.d.=not detected)

Dc measurements

The samples were measured in form of ground and pressed crystalline powders wrapped in Teflon. The mass of the samples was of the order of tens of mg. For the dc measurements and the ac measurements at low frequency, a Quantum Design MPMS SQUID magnetometer (0.1 Hz - 1 kHz) was used. For the ac measurements at high frequency, Quantum Design PPMS equipped with AC susceptibility probe (10 Hz- 10 kHz) was used.

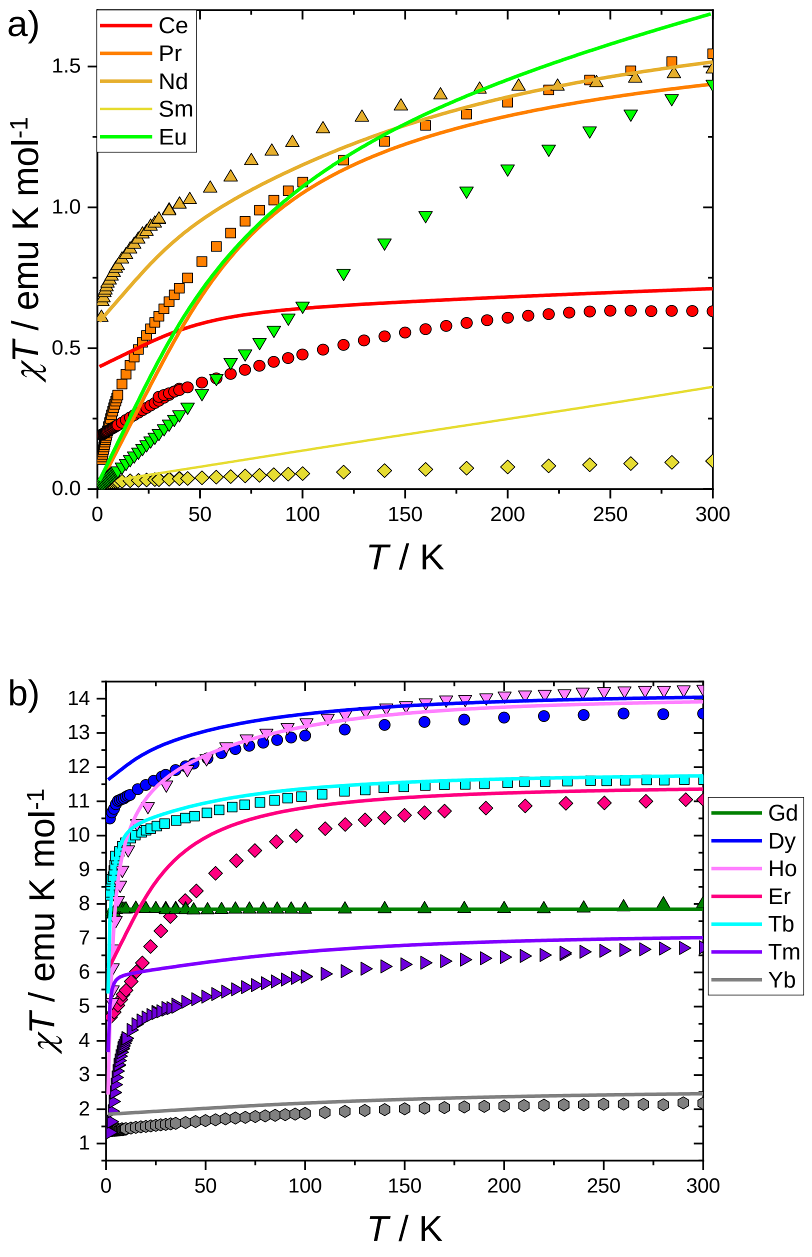

Figure S13. χT vs T curves for all investigated derivatives: a) early lanthanides and b) late lanthanides. Symbols are the experimental points and lines are the *ab initio* simulations.

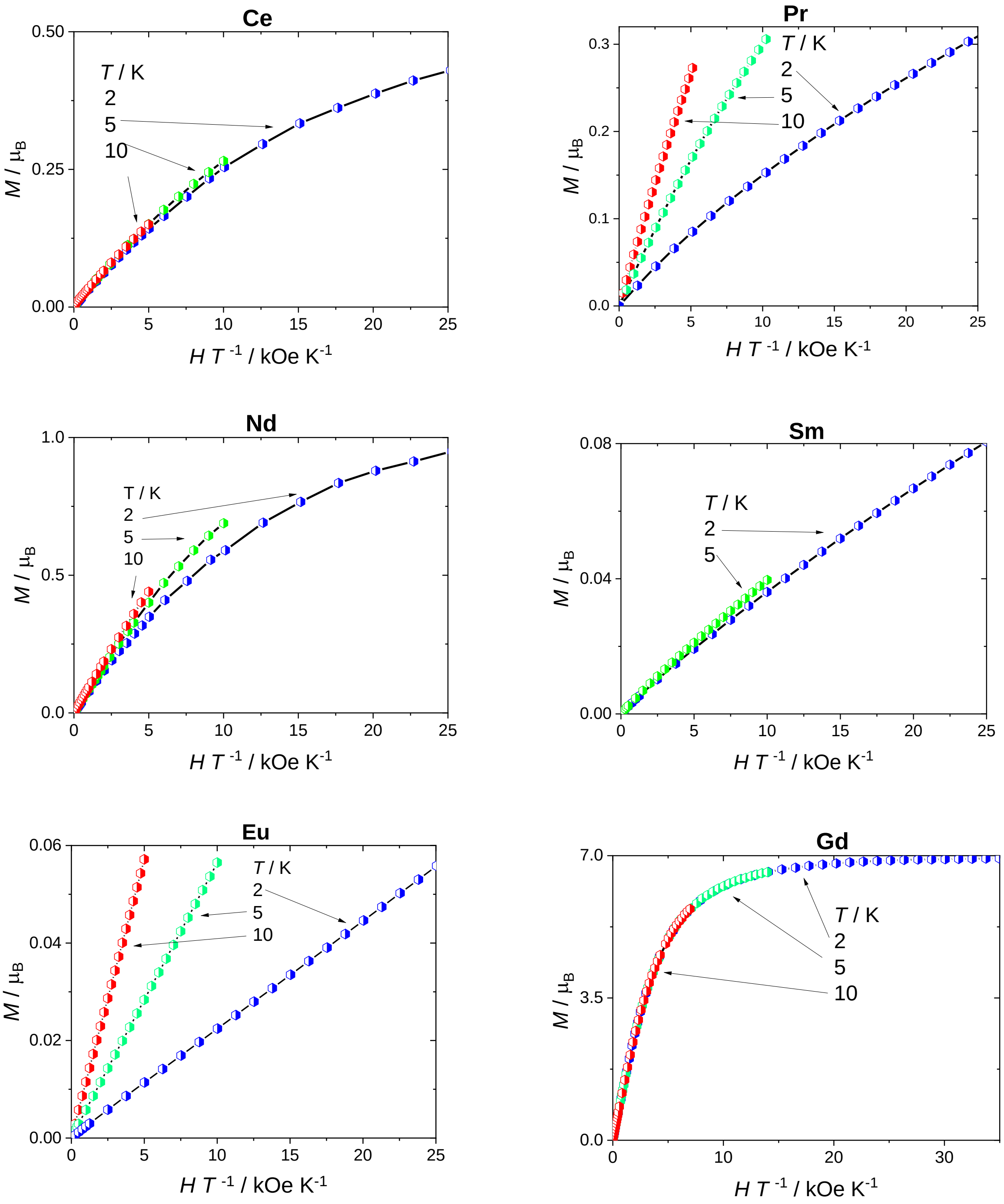

Figure S14. Magnetization curves at different temperatures for all the derivatives not previously measured.[1, 4]

Ac measurements

Cerium

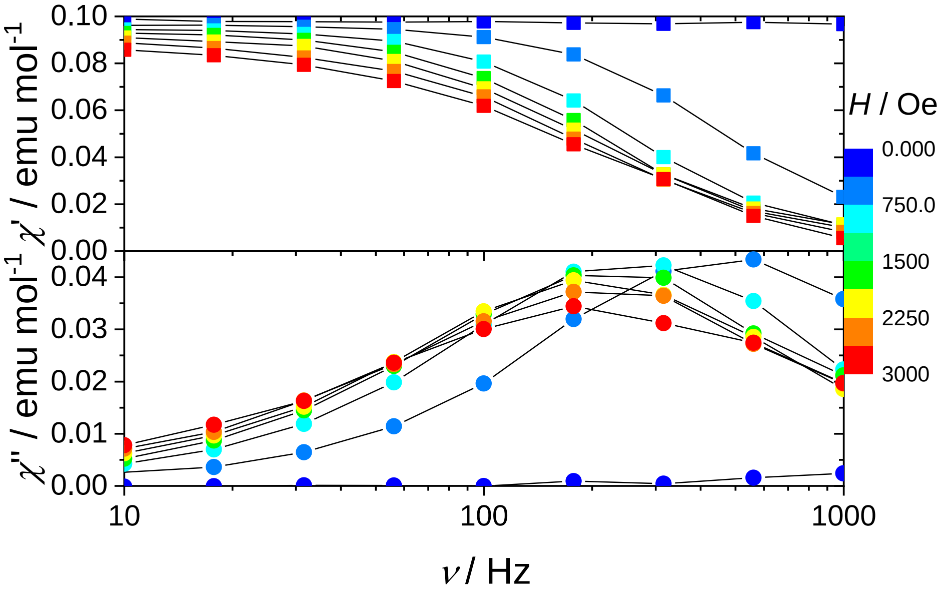

Figure S15. Real (χ') and imaginary (χ'') components of the susceptibility at T = 2K. The field was varied according to the color scale.

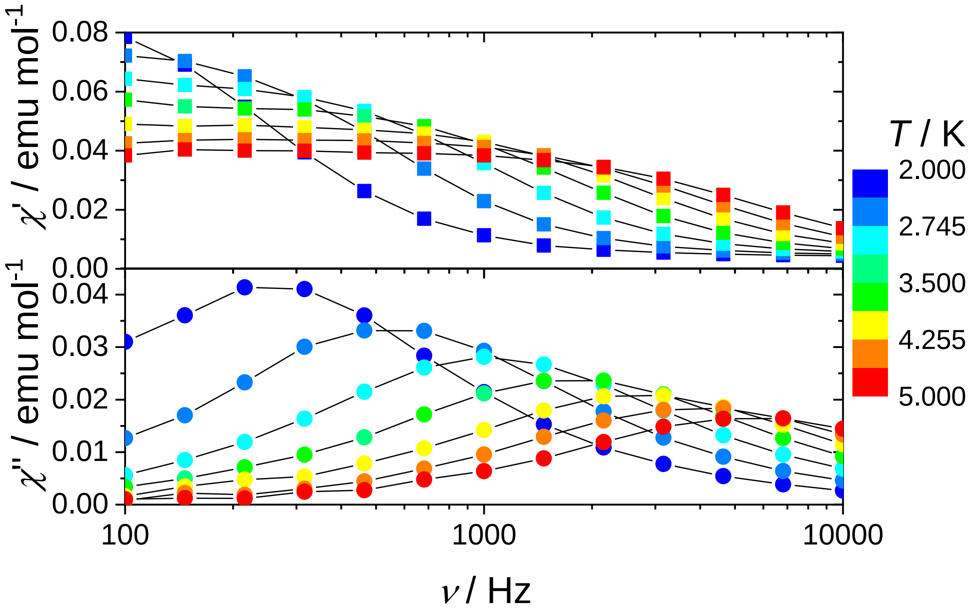

Figure S16. Real (χ') and imaginary (χ'') components of the susceptibility at H = 1000 Oe. The temperature was varied according to the color scale.

Neodymium

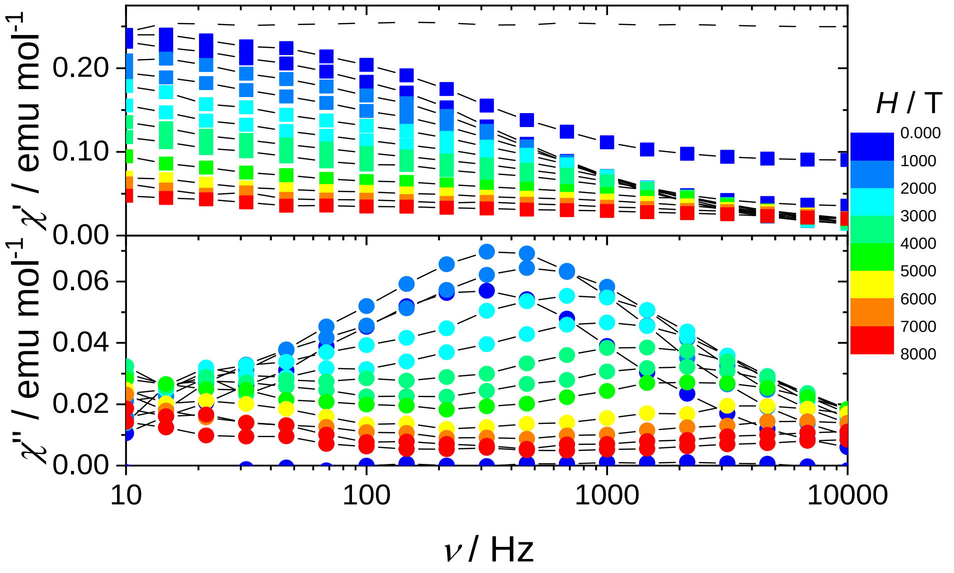

Figure S17. Real (χ') and imaginary (χ'') components of the susceptibility at T = 2 K. The field was varied according to the color scale.

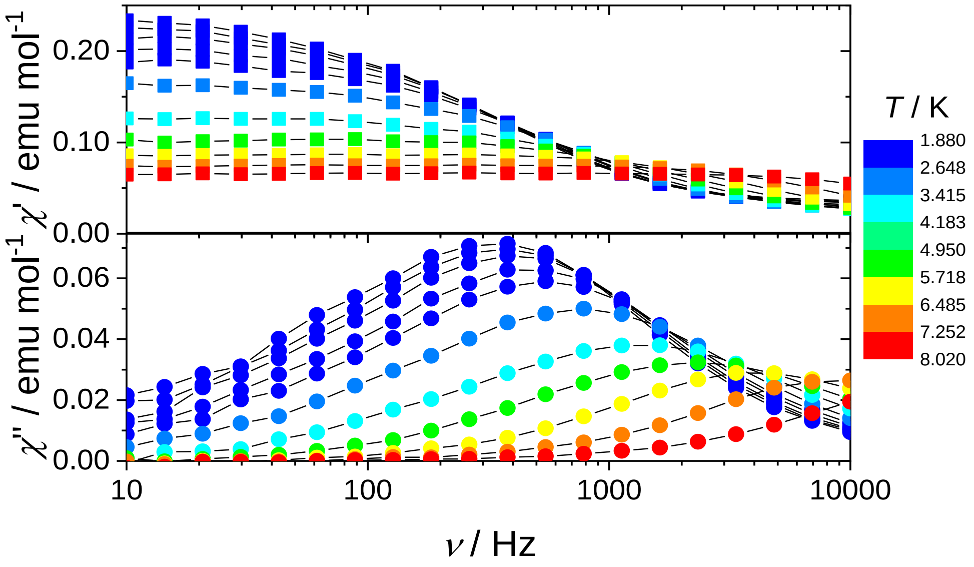

Figure S18. Real (χ') and imaginary (χ'') components of the susceptibility at H = 1000 Oe. The temperature was varied according to the color scale.

Samarium

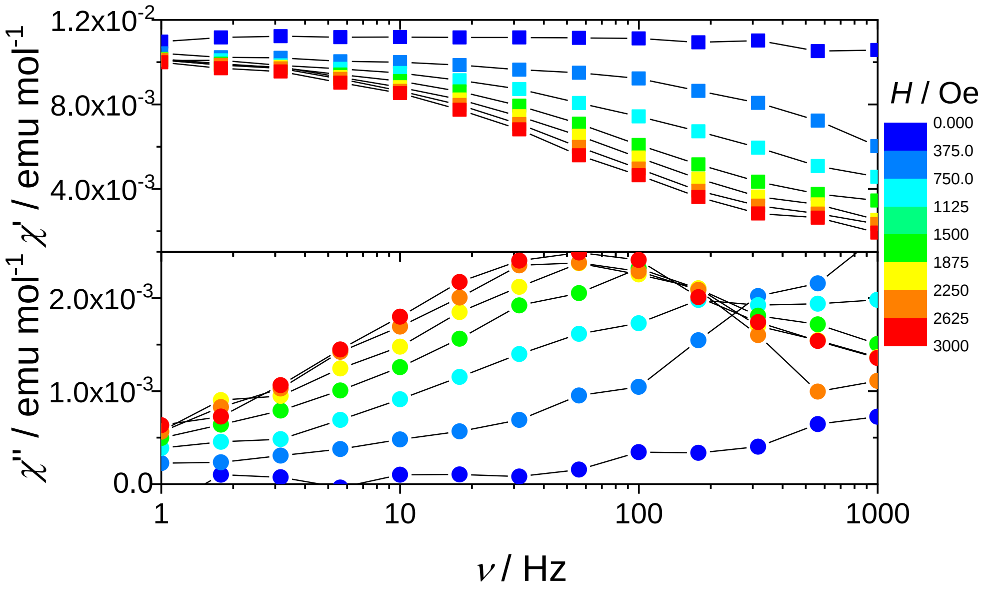

Figure S19. Real (χ') and imaginary (χ'') components of the susceptibility at T = 2 K. The field was varied according to the color scale.

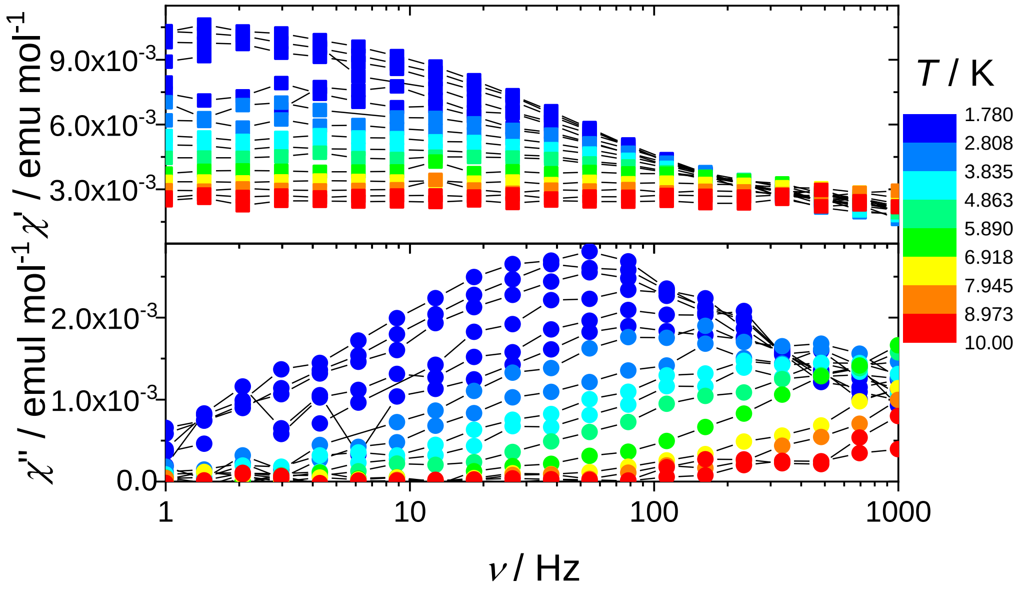

Figure S20. Real (χ') and imaginary (χ'') components of the susceptibility at H = 3000 Oe. The temperature was varied according to the color scale.

Gadolinium

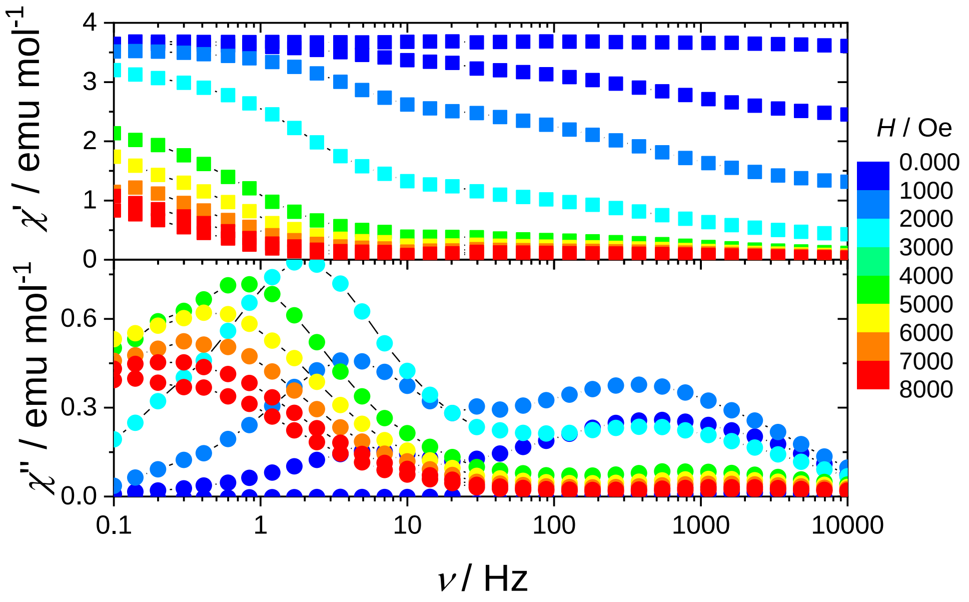

Figure S21. Real (χ') and imaginary (χ'') components of the susceptibility at T = 2 K for the **Gd** derivative. The field was varied according to the color scale.

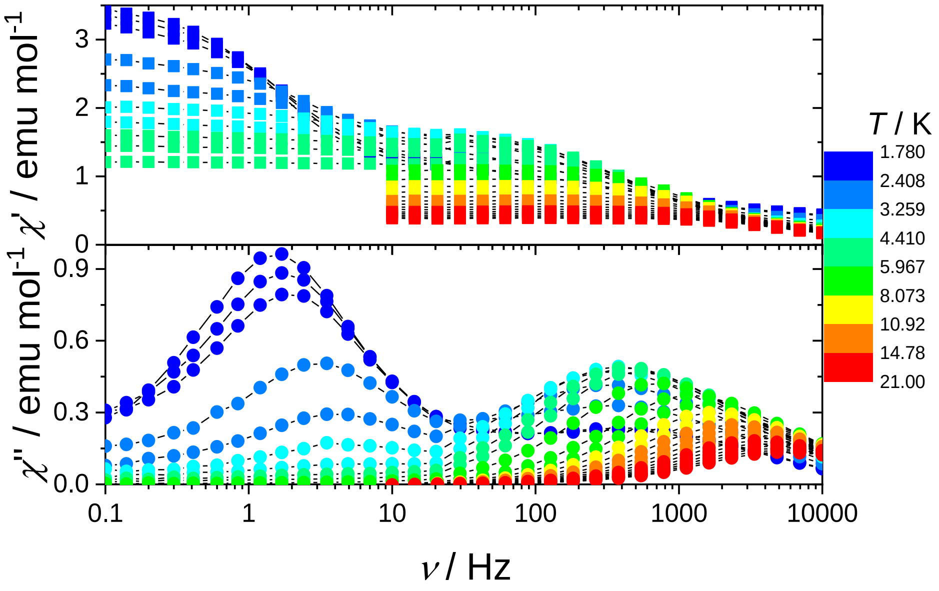

Figure S22. Real (χ') and imaginary (χ'') components of the susceptibility at H = 2000 Oe for the **Gd** derivative. The temperature was varied according to the color scale.

Yttrium-Gadolinium

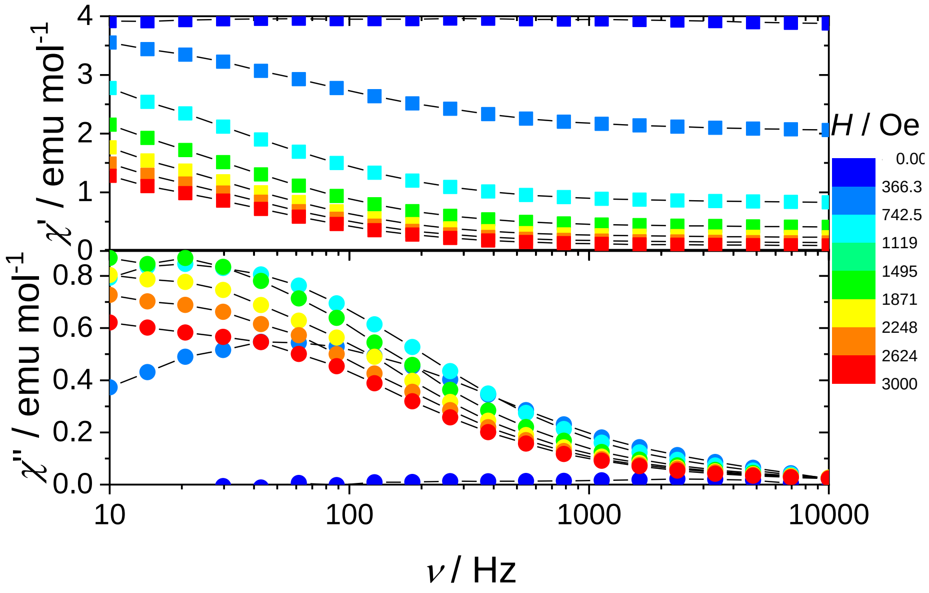

Figure S23. Real (χ') and imaginary (χ'') components of the susceptibility at *T* = 2 K for the **YGd** derivative. The field was varied according to the color scale.

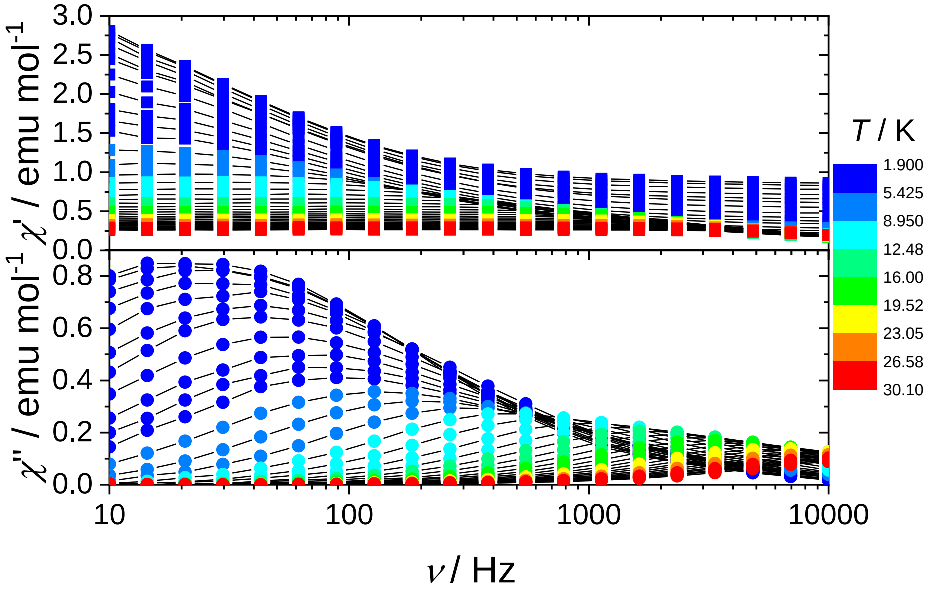

Figure S24. Real (χ') and imaginary (χ'') components of the susceptibility at *H* = 1000 Oe for the **YGd** derivative. The temperature was varied according to the color scale.

Susceptibility - dc vs ac measurements

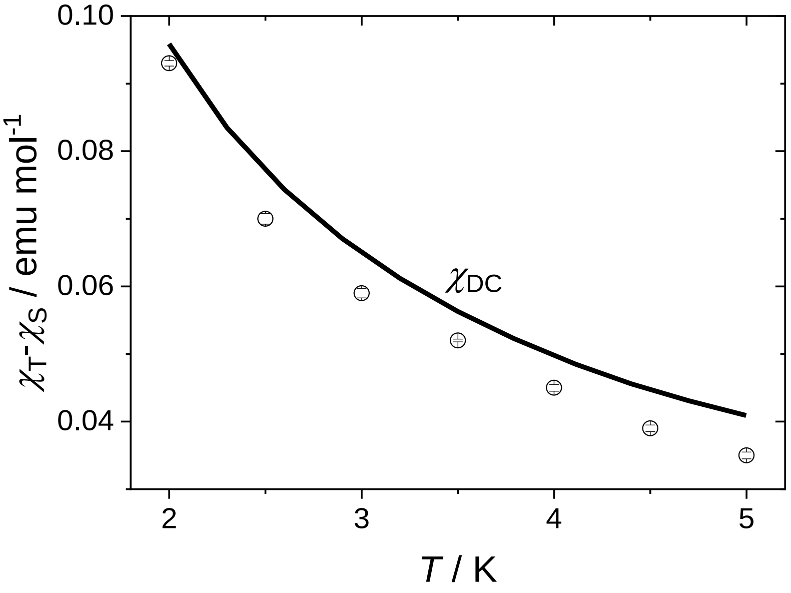

Figure S25. Fraction of the susceptibility that follows the slow relaxation for **Ce**, estimated from the difference between χ_{dc} and the χ_T-χ_S extracted from the ac fits.

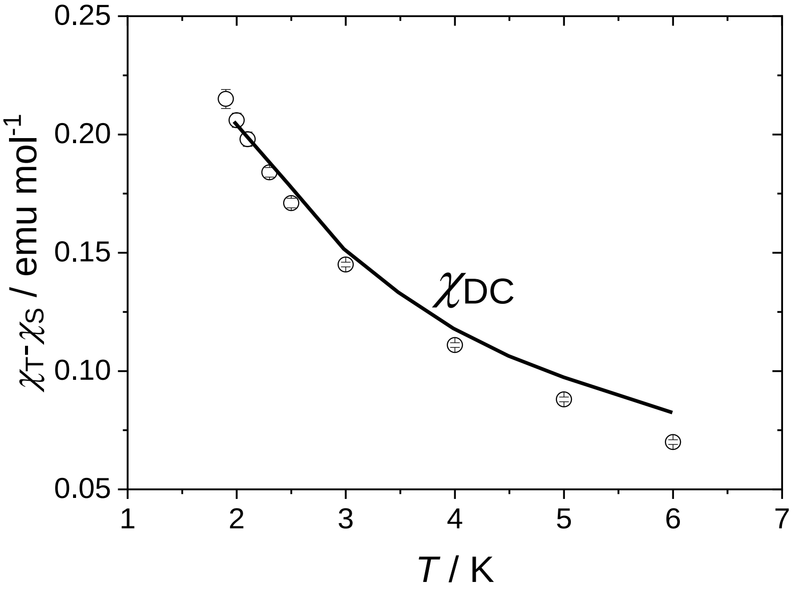

Figure S26. Fraction of the susceptibility that follows the slow relaxation for **Nd**, estimated from the difference between χ_{DC} and the χ_T-χ_S extracted from the ac fits.

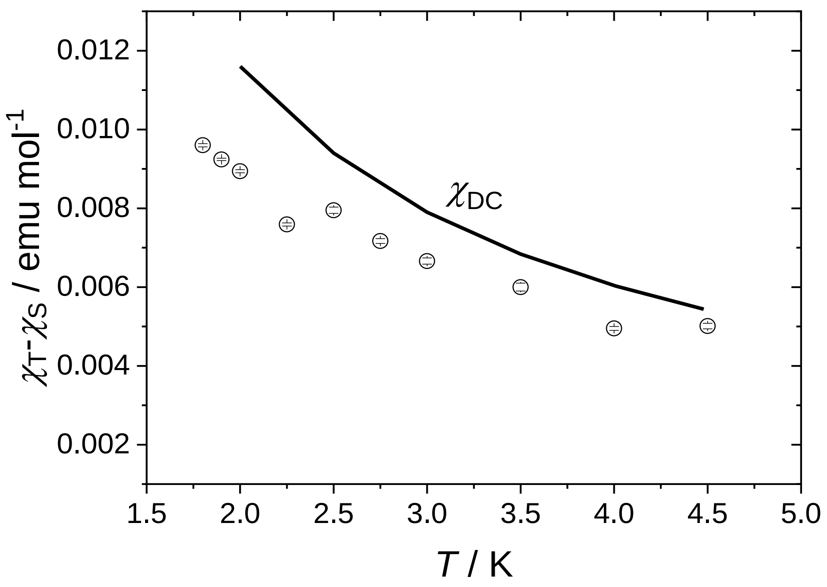

Figure S27. Fraction of the susceptibility that follows the slow relaxation for **Sm**, estimated from the difference between χ_{DC} and the χ_T-χ_S extracted from the ac fits.

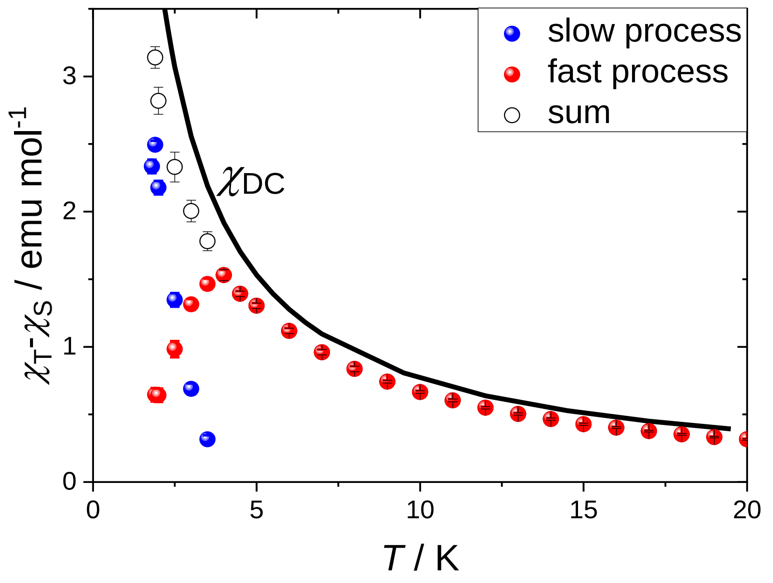

Figure S28. Fraction of the susceptibility that follows the slow relaxation for **Gd**, estimated from the difference between χ_{DC} and the χ_T-χ_S extracted from the ac fits.

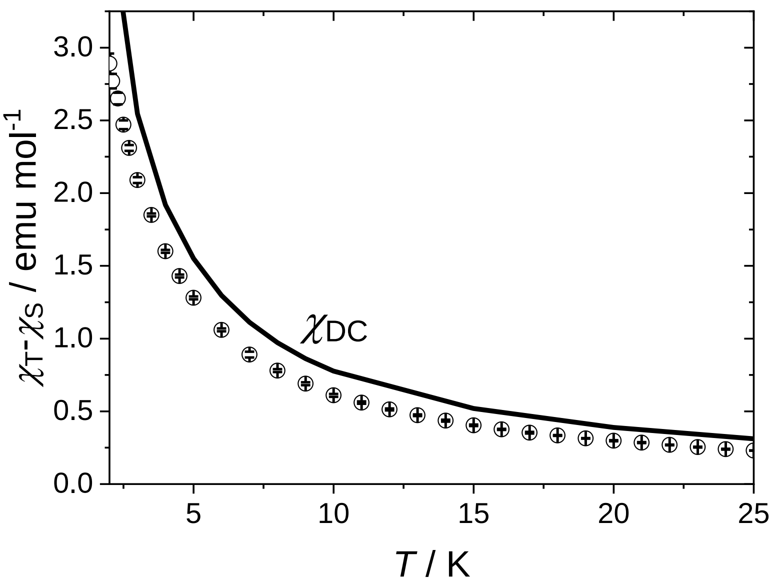

Figure S29. Fraction of the susceptibility that follows the slow relaxation for **YGd**, estimated from the difference between χ_{DC} and the χ_T-χ_S extracted from the ac fits.

Relaxation times

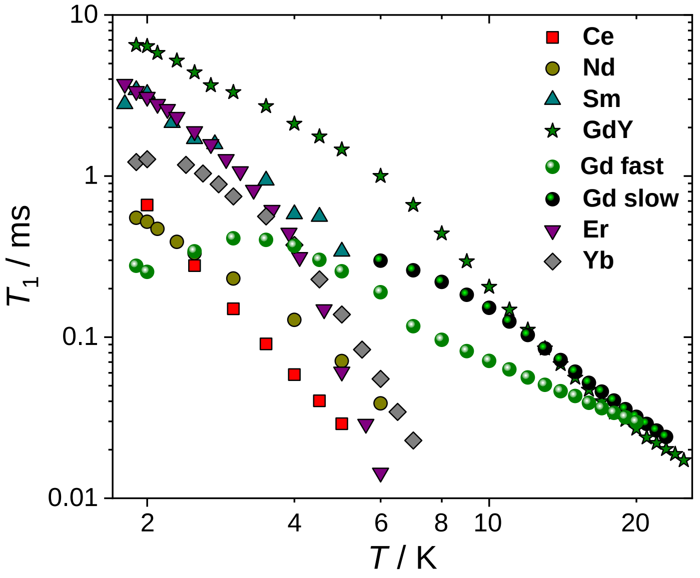

Figure S30. Relaxation time vs temperature for all the in-field SMM of the series except **Dy**. Data of **Er** and **Yb** are taken literature.[5] Fields: **Ce**, **Nd**, **Er**, **Yb**, **GdY** = 0.1 T, **Gd** = 0.2 T, **Sm** = 0.3 T.

Ab initio model

All the calculations in this study were performed on experimentally determined structures (the only exception being the radioactive **Pm**, for which the structure of **Sm** was used). The position of the water's hydrogen atoms was kept fixed on the positions optimized for **Dy** considering the interactions with neighboring chemical groups, as previously discussed.[6] Indeed, analyzing the crystal packing along the whole structural series, the oxygens atoms framework around the apical water molecule is maintained for all the derivatives (see Crystallographic section). Second-order Douglas-Kroll-Hess Hamiltonian has been employed in all the calculations to consider scalar relativistic corrections.

All the calculations were performed with MOLCAS 8.1 Quantum Chemistry Software Package. The energy ladder of the electronic states for every lanthanide ion have been computed within the CASSCF/CASSI-SO method. The employed basis sets and contractions are shown in Table S2. The chosen active space for the lanthanides consists of the unpaired electrons in the seven 4f -orbitals of the lanthanide ion in the oxidation state +3: CAS (N,7), where N is the number of 4f electrons in Ln(III) ions. Due to hardware limitations, only the states with the highest spin multiplicity for each lanthanide, excluded Gd, were computed and included in the following spin-orbit calculation: 7 doublets for Ce, 21 triplets for Pr, 35 quadruplets for Nd, 21 sextuplets for Sm, 7 septuplets for Eu, 7 septuplets for Tb, 21 sextuplets for Dy, 35 quintuplets for Ho, 35 quadruplets for Er, 21 triplets for Tm, 7 doublets for Yb. The choice of considering only the ground state multiplets for the CASSI spin-orbit coupling is based on our experience on DyDOTA complex and on other complexes containing lanthanide ions, for which the inclusion of other multiplets did not substantially improve the solution.[7-8] For Gadolinium were considered 1 octuplet, 48 sextuplets and 215 quadruplets. The *g* and susceptibility tensors for each ion were computed with the SINGLE_ANISO module. For Kramers' ions, the magnetic anisotropy was investigated within the pseudospin framework and their anisotropy axes were calculated with a pseudospin S = 1/2. For ions with even number of electrons the principal directions of the magnetic anisotropy were extrapolated diagonalizing the susceptibility tensor at 2 K.

**Atom**	**Label**	**Primitives**	**Contraction**
Ln	VTZP	[25s22p15d11f4g2h]	[8s7p5d3f2g1h]
Na	VDZ	[17s12p5d4f2g]	[4s3p]
N	VTZP	[14s9p4d3f2g]	[4s3p2d1f]
O	VTZP	[14s9p4d3f2g]	[4s3p2d1f]
C	VDZP	[14s9p4d3f2g]	[3s2p1d]
H	VDZ	[8s4p3d1f]	[2s]

Table S5. Basis sets and contractions employed for the CASSCF calculations.

Ab initio results

	χ_{xx}	χ_{yy}	χ_{zz}
**Ce**	0.0245	0.20555	0.4268
**Pr**	0.0012	0.01685	0.0247
**Nd**	0.0092	0.3684	0.52665
**Pm**	0.0031	0.00605	0.04435
**Sm**	0.00315	0.00485	0.0277
**Eu**	0.00495	0.0054	0.03465
**Gd**	3.2531	4.20455	4.29945
**Tb**	0.0198	0.79155	10.5855
**Dy**	0.02755	0.1053	17.38085
**Ho**	0.024	0.83415	3.77895
**Er**	0.33585	0.45645	8.5045
**Tm**	0.01195	0.0125	7.5724
**Yb**	0.0053	0.011	2.77265

Table S6. Principal values of the susceptibility tensors (emu mol^{-1}) calculated at T = 2 K *ab initio*. The z axis corresponds to the highest susceptibility axis for an easy comparison.

Ln	***g*-tensor**		Orientations of main magnetic axes		
		Main Values	a	b’	c*
**Ce**	g_x	3.001	0.639814	0.334825	0.691759
	g_y	2.063	-0.230790	0.942270	-0.242617
	g_z	0.719	-0.733058	-0.004421	0.680152
**Nd**	g_x	3.319	0.670934	0.148376	0.726520
	g_y	2.759	-0.070829	0.988120	-0.136392
	g_z	0.350	-0.738126	0.040051	0.673473
**Sm**	g_x	0.184	-0.323967	0.879262	-0.349203
	g_y	0.265	-0.605347	-0.476314	-0.637715
	g_z	0.764	-0.727049	0.004790	0.686569
**Dy**	g_x	0.255	0.696343	0.027550	-0.717180
	g_y	0.668	0.216884	0.944468	0.246864
	g_z	19.238	0.684155	-0.327447	0.651698
**Er**	g_x	1.966	0.658552	-0.430484	0.617246
	g_y	2.565	0.206304	0.892074	0.402047
	g_z	13.430	-0.723704	-0.137429	0.676288
**Yb**	g_x	0.094	0.513134	-0.634873	0.577606
	g_y	0.358	0.437125	0.772449	0.460700
	g_z	7.688	-0.738658	0.016085	0.673889

Table S7. Main values and orientations of the ground Kramers’doublet **g** tensor for the Kramers’ ions.

Ln	Main Values of the Susceptibility Tensor at 2 K (cm^3mol^{-1})		Orientations of main magnetic axes		
			a	b’	c*
**Pr**	χ_{xx}	0.02469	-0.671039	-0.017267	-0.741221
	χ_{yy}	0.01684	-0.027448	-0.998465	0.048109
	χ_{zz}	0.00122	-0.740914	0.052628	0.669535
**Pm**	χ_{xx}	0.00311	0.641850	-0.273504	0.716397
	χ_{yy}	0.00606	0.169503	0.961725	0.215300
	χ_{zz}	0.04433	-0.747862	-0.016759	0.663642
**Eu**	χ_{xx}	0.00497	-0.682871	-0.100989	-0.723525
	χ_{yy}	0.00537	0.046287	-0.994390	0.095110
	χ_{zz}	0.03466	-0.729072	0.031458	0.683714
**Tb**	χ_{xx}	0.01981	-0.747231	-0.013196	0.664434
	χ_{yy}	0.79157	-0.207955	0.954235	-0.214918
	χ_{zz}	10.58597	-0.631190	-0.298766	-0.715778
**Ho**	χ_{xx}	0.02398	-0.741485	-0.002298	0.670965
	χ_{yy}	0.83416	-0.054725	0.996870	-0.057062
	χ_{zz}	3.77896	0.668734	0.079029	0.739290
**Tm**	χ_{xx}	0.01193	-0.448063	-0.755615	-0.477793
	χ_{yy}	0.01251	0.496529	-0.654766	0.569860
	χ_{zz}	7.57242	-0.743437	0.018095	0.668561

Table S8. Main values and orientations of the susceptibility tensor times the temperature at 2 K ($cm^3\ mol^{-1}$) for the ions with even number of electrons.

	Ce	Pr	Nd	Pm	Sm	Gd	Tb	Dy	Ho	Er	Tm	Yb
B_2^2	4.8943e+01	1.7310e+01	-3.0955e-01	3.7937e-01	-2.3450e+00	-3.0308e-02	4.4448e+00	4.1479e+00	-5.6475e-02	1.4329e-01	-3.4598e-01	1.1709e-01
B_2^1	3.3899e+00	1.3898e+00	2.6030e-01	-5.0620e-01	4.9486e+00	1.2602e-04	6.1730e-01	2.3428e-01	7.9268e-02	5.1235e-01	-1.5425e-01	-1.2829e-01
B_2^0	-1.9407e+01	-6.0564e+00	3.6865e+00	-3.3255e+00	-2.0275e+01	-1.1788e-02	-2.7383e+00	-1.7122e+00	1.0407e+00	-1.1644e+00	-4.8358e+00	-1.4579e+01
B_2^{-1}	-7.9085e-01	2.5908e+00	5.2041e-01	7.7000e-01	-1.0751e+01	1.9652e-06	1.8234e-01	3.0499e-01	-3.6570e-02	7.9392e-01	3.2082e-01	-2.7785e-01
B_2^{-2}	-4.0565e-01	-4.3739e-01	-1.0731e-02	-1.2373e-01	-6.0203e-01	-3.9906e-05	-5.8372e+00	-2.4430e-02	-3.8208e-02	-1.1129e-01	2.5651e-01	-1.3914e+00
B_4^4	-1.4810e+00	-1.0472e-01	7.3177e-02	2.7200e-01	1.7676e+00	-9.3240e-06	3.4684e-04	-8.0469e-03	1.7615e-02	2.1463e-02	7.5291e-02	-5.0440e-02
B_4^3	1.3593e+00	-6.0034e-02	-2.5838e-02	9.0393e-02	2.0494e+00	1.1008e-05	-6.4158e-02	1.6371e-02	2.1813e-03	7.0454e-03	-1.3532e-03	-8.1269e-02
B_4^2	-4.2561e+00	-1.3090e-02	1.0055e-02	-9.1140e-03	-3.1064e-01	2.6694e-06	-2.7050e-02	-1.9042e-02	9.3682e-04	-7.9590e-04	1.4699e-03	-2.0026e-02
B_4^1	-1.2986e-01	1.2857e-02	-8.8565e-03	1.4310e-02	-7.0467e-01	-1.4576e-06	-2.2757e-02	4.7052e-03	-5.9786e-04	2.6168e-04	-2.5764e-03	4.7586e-04
B_4^0	-7.5618e-01	-2.0327e-02	-5.5988e-03	1.2990e-03	-4.2519e-02	-4.0560e-07	-5.1654e-03	-5.1991e-03	-2.4804e-04	1.8072e-04	6.0154e-04	-8.7960e-03
B_4^{-1}	-2.7332e+00	7.1480e-01	-9.1670e-04	-1.9429e-02	7.6838e-01	-3.2584e-05	-3.7844e-02	4.8983e-03	2.5565e-04	-1.2658e-03	-1.5329e-03	3.0269e-02

B_4^{-2}	-4.4440e-01	1.8382e-02	-4.0596e-03	2.4000e-02	-1.6391e-01	-3.0810e-06	2.6784e-02	-1.4254e-03	9.7819e-05	1.0322e-03	-4.1728e-03	-4.1279e-02
B_4^{-3}	-4.2306e+00	1.1728e+00	-3.8031e-02	-1.0539e-02	1.6926e+00	-4.1417e-05	-1.4637e-02	-5.9267e-04	-4.1436e-03	-1.8368e-02	1.5515e-02	1.5797e-01
B_4^{-4}	-2.9853e-01	-2.2742e-03	-2.4787e-01	1.2590e-01	-5.3774e-01	-3.9635e-06	6.3961e-03	-1.5092e-03	-3.7299e-03	-1.3499e-02	4.2579e-04	7.9403e-01
B_6^{6}		-2.8121e-03	-1.5223e-03	1.9486e-03		3.8264e-10	2.2060e-04	2.2341e-05	5.3290e-05	-1.2072e-04	-1.0170e-05	-4.2386e-03
B_6^{5}		-3.1093e-03	3.6396e-03	1.7000e-02		-2.0909e-08	-1.1124e-03	2.5071e-04	2.3271e-04	1.1022e-03	1.7077e-04	-1.1757e-02
B_6^{4}		-3.2674e-02	6.9848e-03	-3.2119e-02		-8.2058e-09	1.0806e-05	-2.9627e-04	-3.0785e-04	-2.1434e-04	1.2731e-03	1.8879e-02
B_6^{3}		3.3676e-03	2.8457e-03	-5.4000e-04		-2.5180e-09	-3.7626e-04	-2.4834e-04	1.2619e-05	1.8730e-04	1.4106e-04	1.6205e-03
B_6^{2}		9.6372e-04	-4.6552e-04	2.3465e-03		1.3456e-09	-6.2109e-06	-9.9083e-06	6.8968e-06	7.0838e-05	9.4085e-05	2.0117e-03
B_6^{1}		4.6659e-03	-3.2156e-04	-7.9950e-04		1.2131e-09	7.2995e-05	-1.0218e-04	-7.5939e-06	-1.1617e-04	9.7807e-05	-4.5012e-03
B_6^{0}		2.3861e-03	-5.6255e-04	1.2582e-03		3.9212e-10	1.3066e-05	1.1057e-05	-2.2223e-05	1.9702e-05	-8.1296e-05	1.9077e-03
B_6^{-1}		-1.4981e-02	1.3855e-03	-6.5731e-03		-8.2902e-09	1.6047e-04	-1.3750e-04	5.4476e-05	-2.0646e-04	-1.3484e-04	-5.3025e-04
B_6^{-2}		-2.0770e-03	-1.0383e-03	4.3198e-04		-2.0390e-09	5.9178e-05	9.3797e-05	-2.5600e-05	6.8230e-05	1.8728e-05	-1.3817e-03
B_6^{-3}		2.6358e-02	2.1201e-03	9.5110e-03		1.8761e-08	-1.3454e-04	2.1301e-04	8.6496e-05	4.2143e-04	1.6956e-04	6.0749e-03
B_6^{-4}		-1.5885e-03	2.2633e-02	1.3973e-02		-2.1944e-09	2.5386e-05	-5.3537e-05	3.2622e-04	4.8568e-04	-8.9379e-04	3.1289e-02
B_6^{-5}		7.9485e-02	-8.9437e-03	1.3700e-02		2.6327e-08	8.2791e-04	7.8168e-04	8.3789e-05	-3.5109e-04	7.2638e-04	-1.5717e-02
B_6^{-6}		3.1036e-03	-2.7719e-03	-4.7100e-03		1.8526e-09	1.7060e-04	-3.4412e-05	-2.3842e-05	-9.8312e-05	-2.2213e-04	3.8050e-03

Table S9. Projection of the CF parameters calculated *ab initio* on the ground *J* multiplet of each Ln (expressed in Stevens notation). For Eu, the ground state is *J* = 0. The parameters are expressed in the orthogonal *ab'c** reference frame.

		I Doublet	II Doublet
**Ce**	**Energy (cm^{-1})**	0	109
	**|±5/2 >**	75.3	23.8
	**|±3/2 >**	10.1	31.5
	**|±1/2 >**	14.6	44.7
**Nd**	**Energy (cm^{-1})**	0	81
	**|±9/2 >**	21.5	32.8
	**|±7/2 >**	39.9	2.8
	**|±5/2 >**	9.1	35.5
	**|±3/2 >**	16.5	14.2
	**|±1/2 >**	13.0	14.7
**Sm**	**Energy (cm^{-1})**	0	266
	**|±5/2 >**	95.1	4.4
	**|±3/2 >**	3.9	86.4

	**|±1/2 >**	1.1	9.2
**Dy**	**Energy (cm^{-1})**	0.00	47
	**|±15/2 >**	94.6	2.9
	**|±13/2 >**	0.0	2.4
	**|±11/2 >**	0.4	3.8
	**|±9/2 >**	0.3	4.8
	**|±7/2 >**	2.4	5.2
	**|±5/2 >**	0.3	15.6
	**|±3/2 >**	1.3	24.5
	**|±1/2 >**	0.7	36.6
**Er**	**Energy (cm^{-1})**	0	41
	**|±15/2 >**	20.3	59.5
	**|±13/2 >**	48.2	16.7
	**|±11/2 >**	11.8	2.6
	**|±9/2 >**	1.9	2.8
	**|±7/2 >**	5.9	4.7
	**|±5/2 >**	5.2	7.4
	**|±3/2 >**	3.9	4.8
	**|±1/2 >**	2.9	1.5
**Yb**	**Energy (cm^{-1})**	0.00	257
	**|±7/2 >**	96.8	0.2
	**|±5/2 >**	0.1	92.4
	**|±3/2 >**	0.1	7.4
	**|±1/2 >**	3.0	0.0

Table S10. *Ab initio* calculated energies and |m_j > percentage components of ground and first excited doublet states arising from the ground *J* multiplet of the **Ln** complexes with odd number of electrons (Kramers' ions). The quantization axis is along the easy axis of the ground doublet.

		I	II	III	IV
**Pr**	**Energy (cm^{-1})**	0	112	145	235
	**|±4 >**	51.7	76.7	1.1	6.2
	**|±3 >**	1.0	7.4	42.0	77.0
	**|±2 >**	14.2	10.1	8.7	1.4
	**|±1 >**	6.4	5.8	48.2	6.7
	**|0 >**	26.6	0.0	0.0	8.7
**Pm**	**Energy (cm^{-1})**	0	41	65	74

	**|±4 >**	67.9	88.4	2.6	7.4
	**|±3 >**	0.1	2.2	83.6	32.5
	**|±2 >**	2.2	9.2	0.1	56.4
	**|±1 >**	1.3	0.2	13.1	3.6
	**|0 >**	28.5	0.1	0.4	0.1
**Tb**	**Energy (cm^{-1})**	0	2	11	15
	**|±6 >**	78.1	92.3	3.0	17.4
	**|±5 >**	1.4	1.9	7.3	3.9
	**|±4 >**	4.7	1.6	1.9	8.1
	**|±3 >**	1.4	1.7	25.2	3.6
	**|±2 >**	8.1	0.5	4.2	36.6
	**|±1 >**	0.4	1.9	58.2	1.9
	**|0 >**	5.8	0.0	0.1	28.4
**Ho**	**Energy (cm^{-1})**	0	8	25	45
	**|±8 >**	41.8	54.2	2.1	13.5
	**|±7 >**	13.8	17.8	11.5	18.7
	**|±6 >**	0.3	0.7	10.7	2.9
	**|±5 >**	19.4	13.4	2.1	9.0
	**|±4 >**	8.4	9.6	24.2	8.8
	**|±3 >**	0.9	0.3	21.0	31.2
	**|±2 >**	10.5	3.5	1.3	4.5
	**|±1 >**	0.3	0.5	26.9	0.4
	**|0 >**	4.7	0.0	0.2	11.0
**Tm**	**Energy (cm^{-1})**	0	2	193	198
	**|±6 >**	97.1	97.8	0.0	0.0
	**|±5 >**	0.0	0.0	93.3	94.8
	**|±4 >**	0.0	0.0	0.5	0.6
	**|±3 >**	0.0	0.0	0.3	0.2
	**|±2 >**	2.8	2.1	0.0	0.0
	**|±1 >**	0.0	0.0	5.8	4.3
	**|0 >**	0.0	0.0	0.0	0.0

Table S11. *Ab initio* calculated energies and $|m_j>$ percentage components of ground and first three excited singlet states arising from the ground J multiplet of the **Ln** complexes with even number of electrons. The quantization axis is along the easy axis of the susceptivity tensor diagonalized at 2 K. Plus and minus $|m_j>$ components have been summed up, thus the sum along the row can go beyond 100% (up to 200%).

CTM simulations using the *ab initio* results

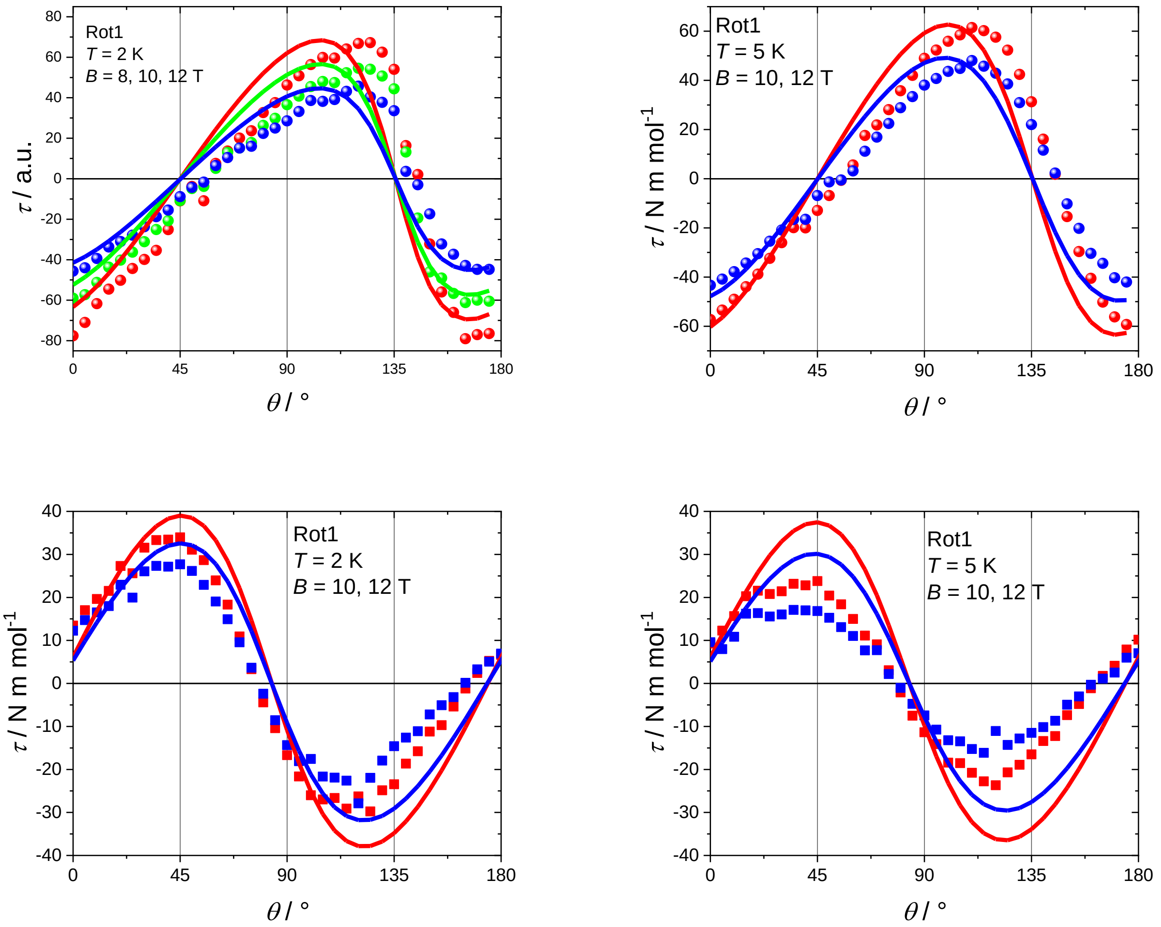

Figure S31. Torque curves obtained at all temperatures and field for **Ce**: Rot1 (dots) and Rot2 (squares). A scaling factor has been applied to the experimental torque due to the incertitude on the crystal's mass.

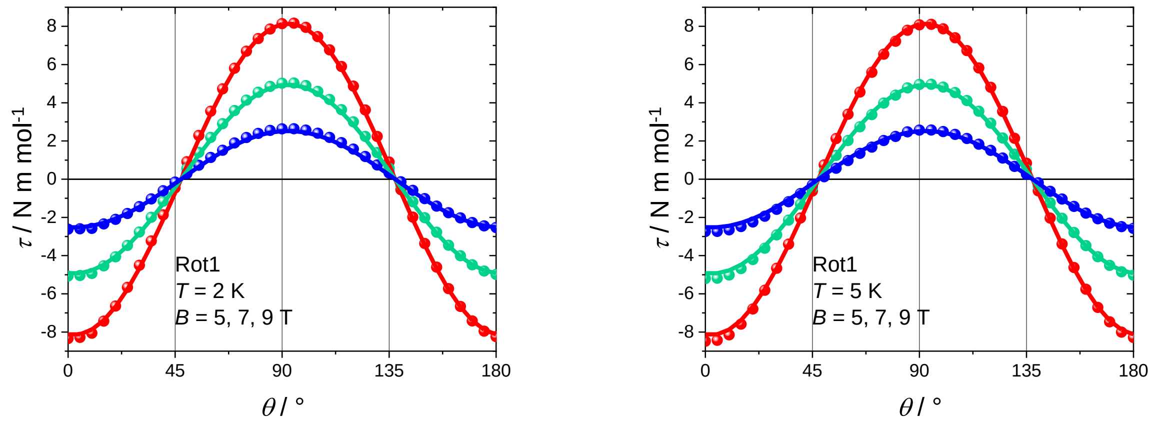

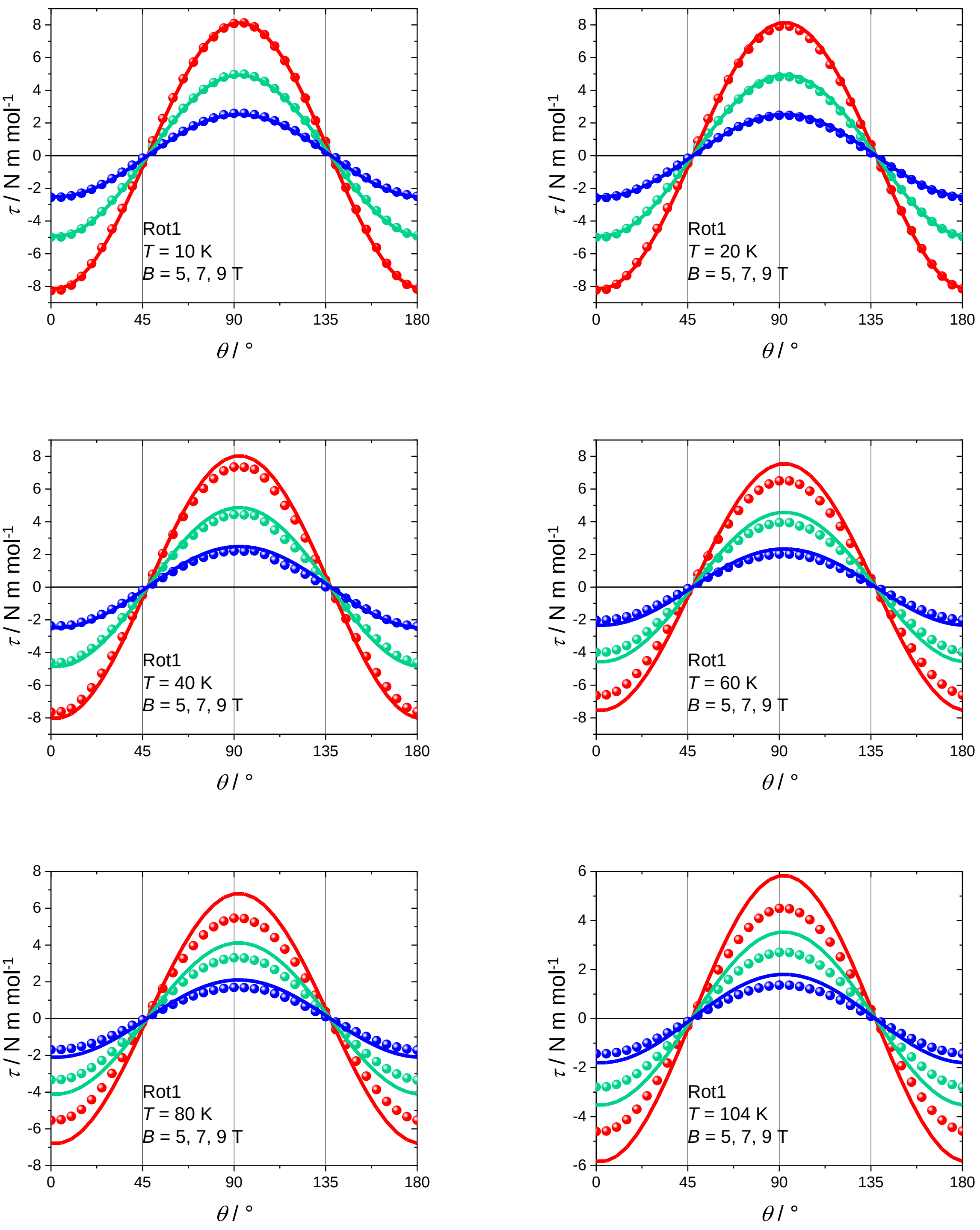

Rot1
T = 10 K
B = 5, 7, 9 T
Rot1
T = 20 K
B = 5, 7, 9 T
Rot1
T = 40 K
B = 5, 7, 9 T
Rot1
T = 60 K
B = 5, 7, 9 T
Rot1
T = 80 K
B = 5, 7, 9 T
Rot1
T = 104 K
B = 5, 7, 9 T
τ / N m mol^{-1}
θ / °

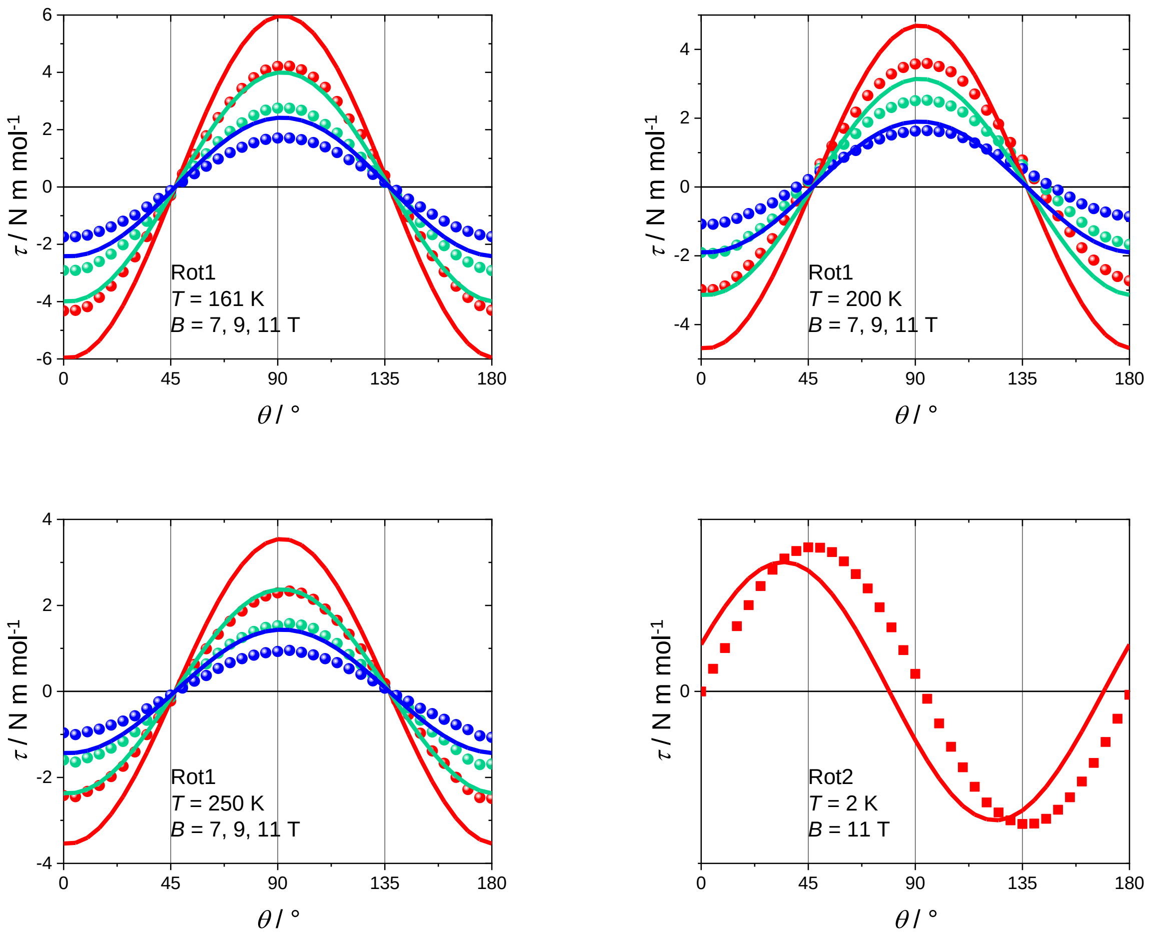

Figure S32. Torque curves obtained at all temperatures and field for **Pr**: Rot1 (dots) and Rot2 (squares). A scaling factor has been applied to the experimental torque due to the incertitude on the crystal's mass.

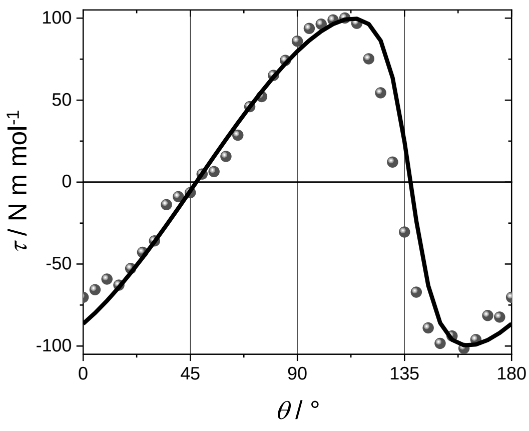

Figure S33. Torque curves obtained at all temperatures and field for **Nd**: Rot1 (dots). A scaling factor has been applied to the experimental torque due to the incertitude on the crystal's mass.

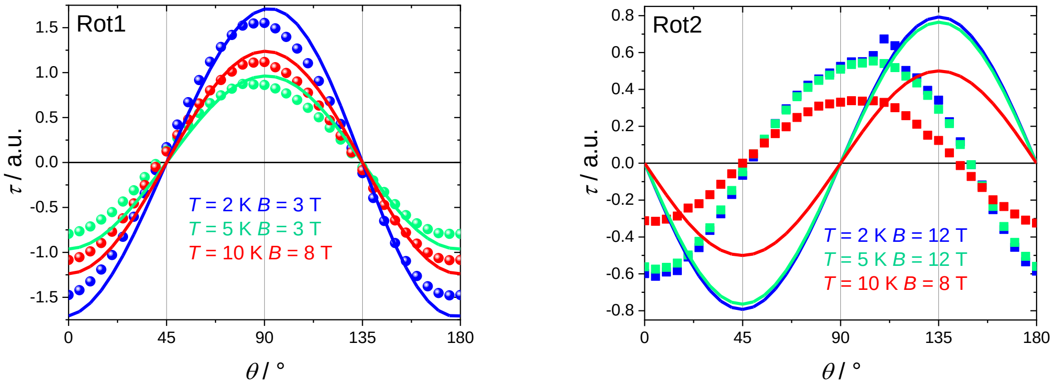

Figure S34. Torque curves obtained at all temperatures and field for **Gd**: Rot1 (dots) and Rot2 (squares). A scaling factor has been applied to the experimental torque due to the incertitude on the crystal's mass.

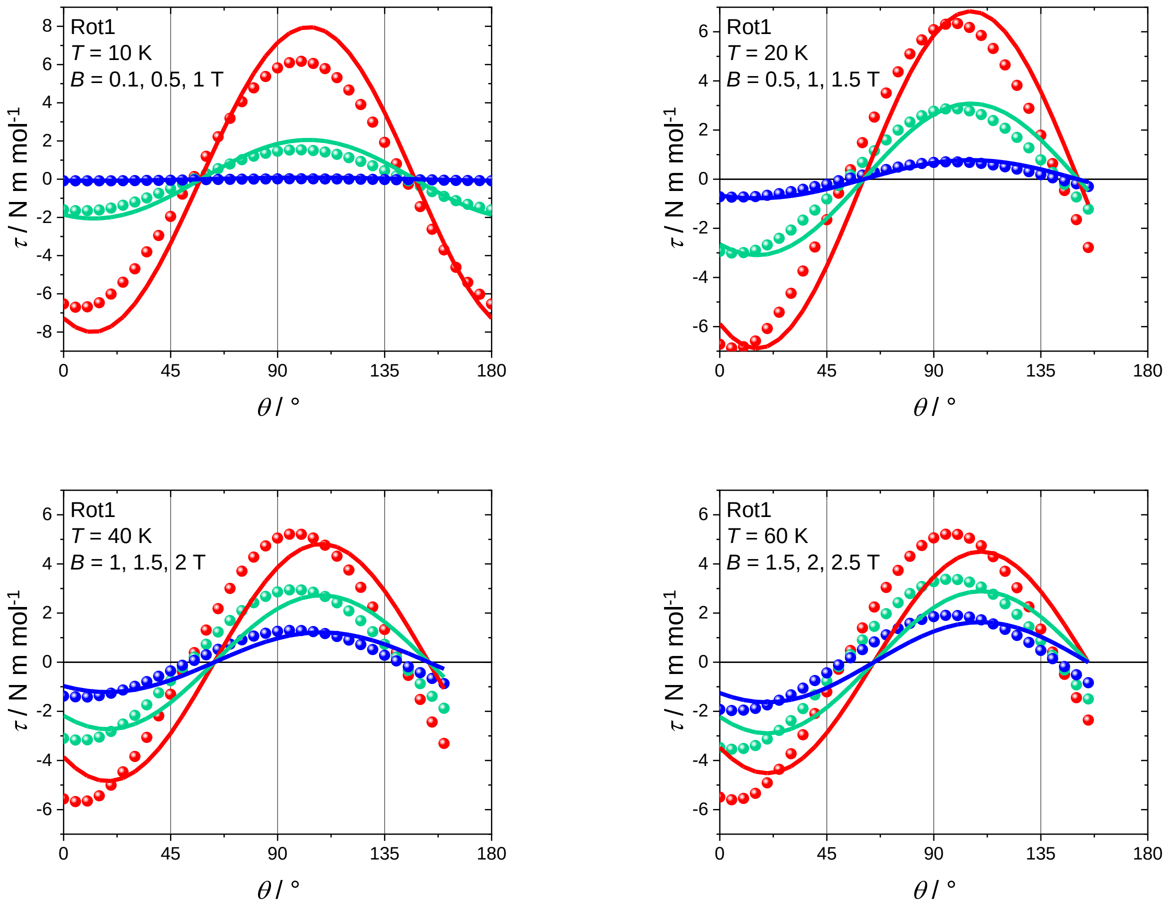

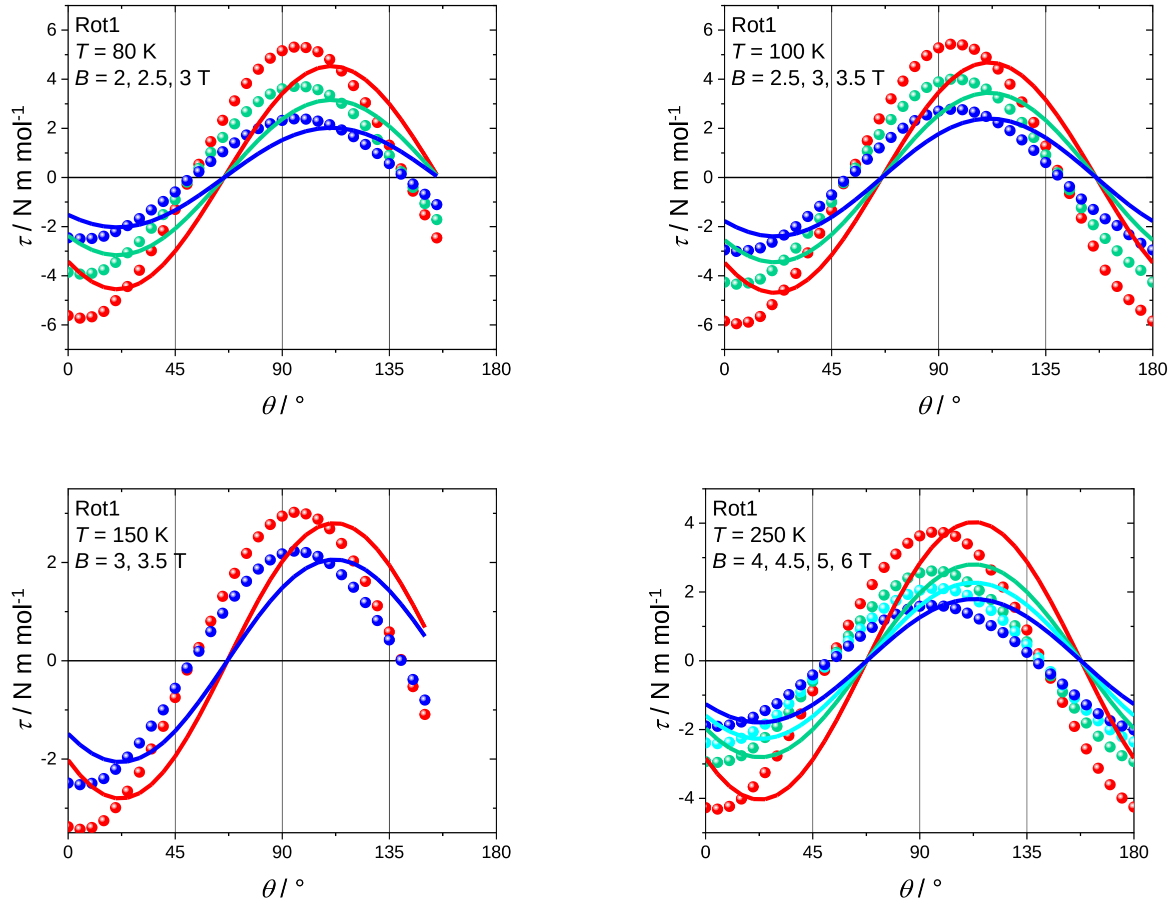

Figure S35. Torque curves obtained at all temperatures and field for **Tb**: Rot1 (dots). A scaling factor has been applied to the experimental torque due to the incertitude on the crystal's mass.

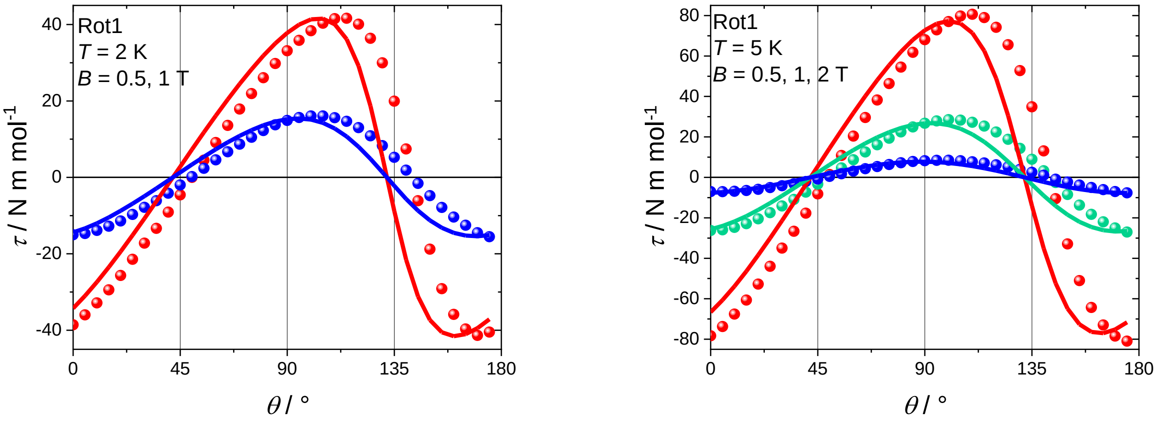

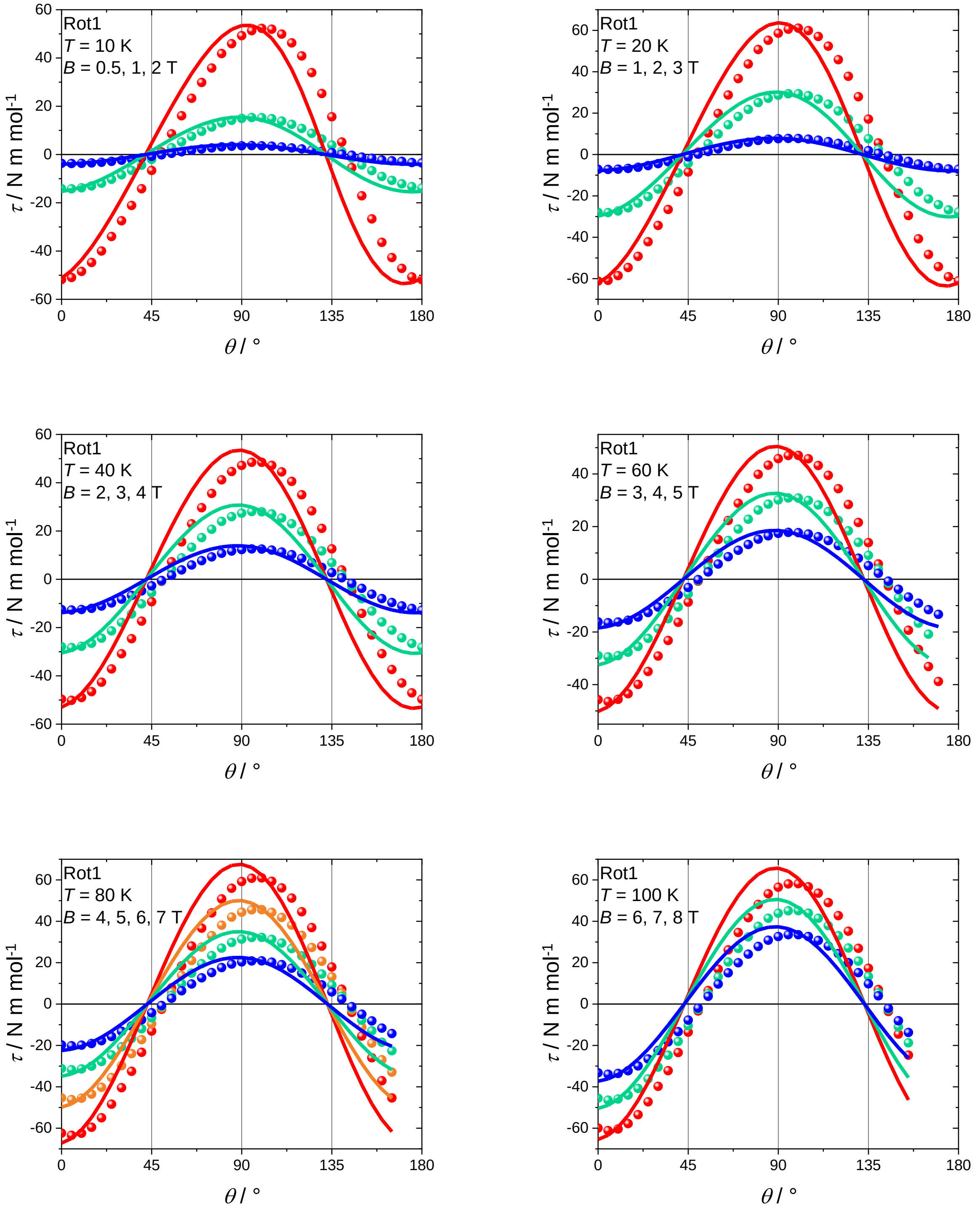
Rot1
T = 10 K
B = 0.5, 1, 2 T
Rot1
T = 20 K
B = 1, 2, 3 T
Rot1
T = 40 K
B = 2, 3, 4 T
Rot1
T = 60 K
B = 3, 4, 5 T
Rot1
T = 80 K
B = 4, 5, 6, 7 T
Rot1
T = 100 K
B = 6, 7, 8 T
τ / N m mol-1
θ / °

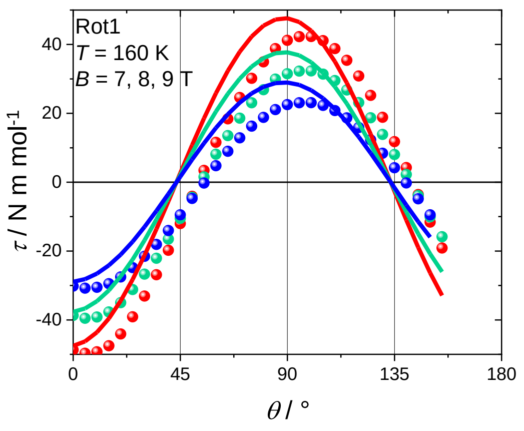

Figure S36. Torque curves obtained at all temperatures and field for **Dy**: Rot1 (dots). A scaling factor has been applied to the experimental torque due to the incertitude on the crystal's mass.

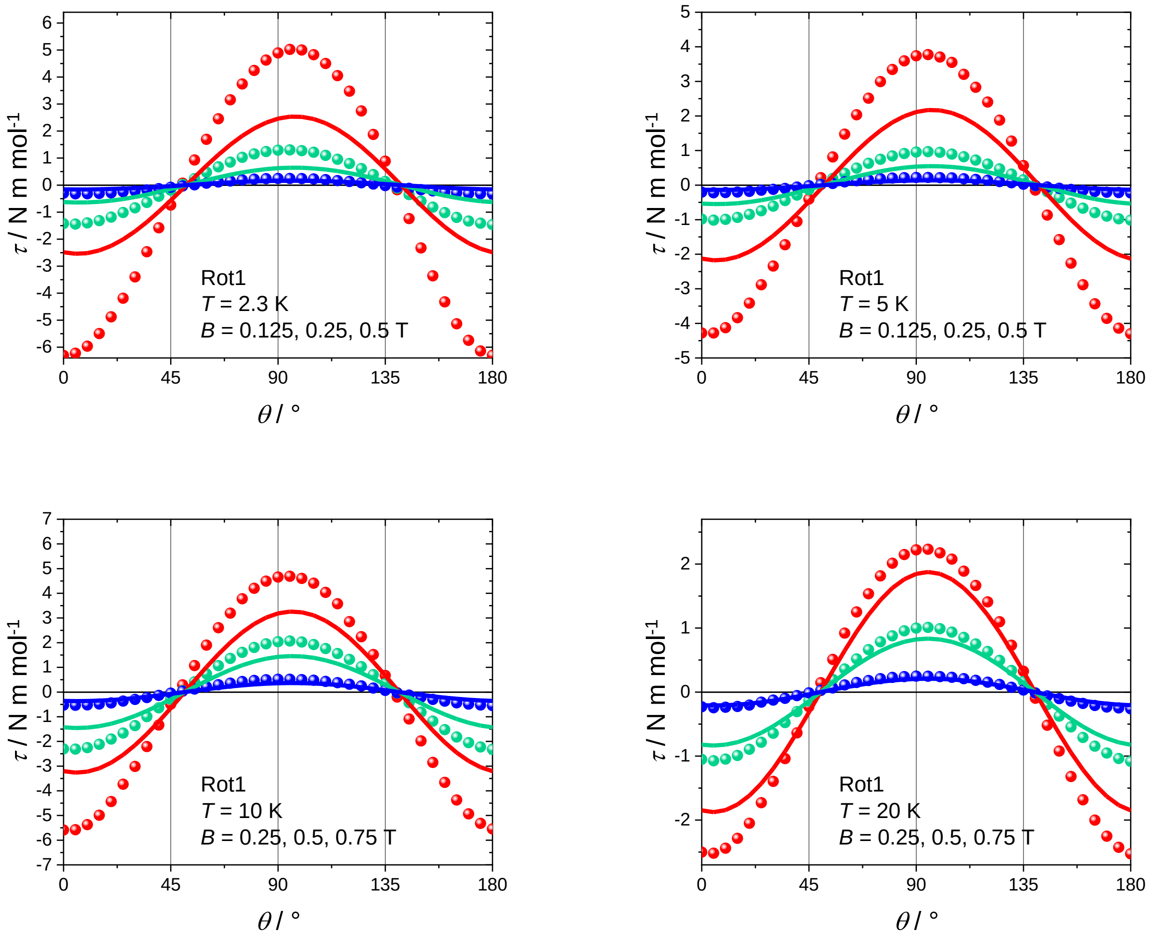

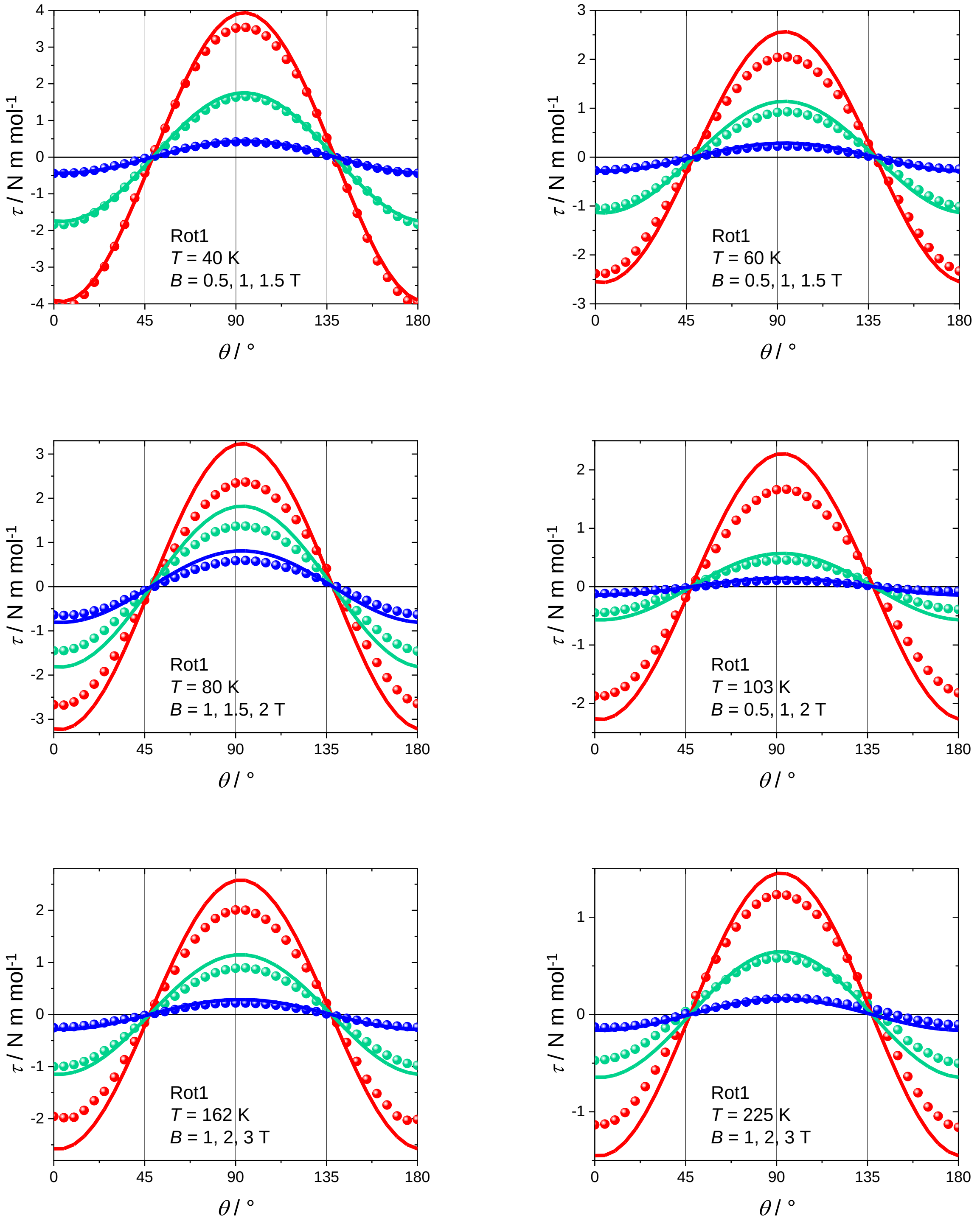

Figure S37. Torque curves obtained at all temperatures and field for **Ho**: Rot1 (dots). A scaling factor has been applied to the experimental torque due to the incertitude on the crystal's mass.

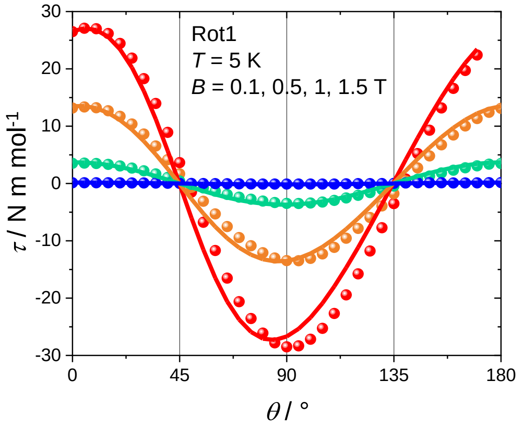
Rot1
T = 5 K
B = 0.1, 0.5, 1, 1.5 T
τ / N m mol-1
θ / °

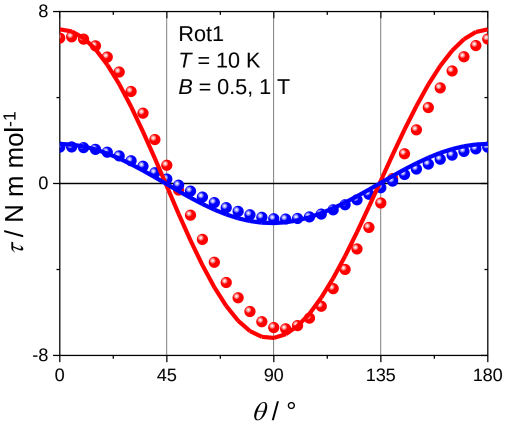
Rot1
T = 10 K
B = 0.5, 1 T
τ / N m mol-1
θ / °

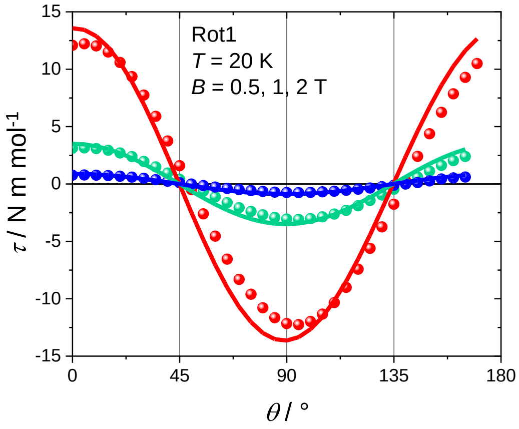
Rot1
T = 20 K
B = 0.5, 1, 2 T
τ / N m mol-1
θ / °

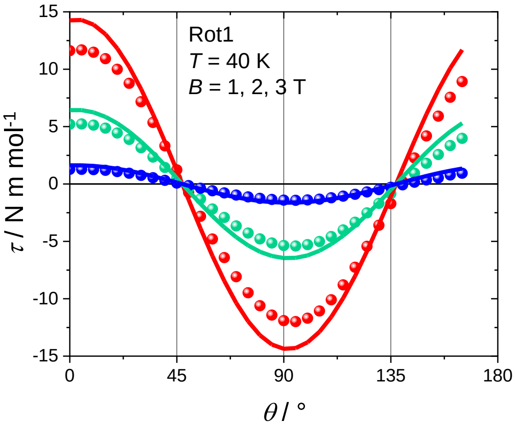
Rot1
T = 40 K
B = 1, 2, 3 T
τ / N m mol-1
θ / °

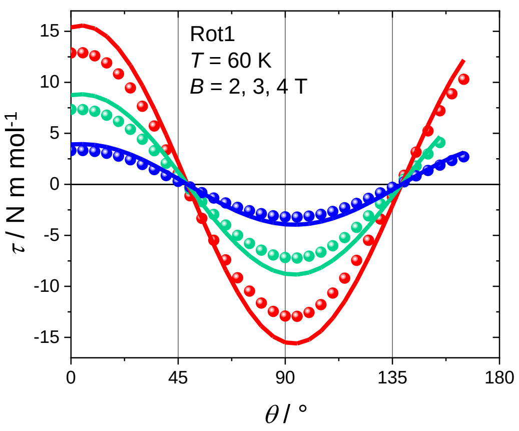
Rot1
T = 60 K
B = 2, 3, 4 T
τ / N m mol-1
θ / °

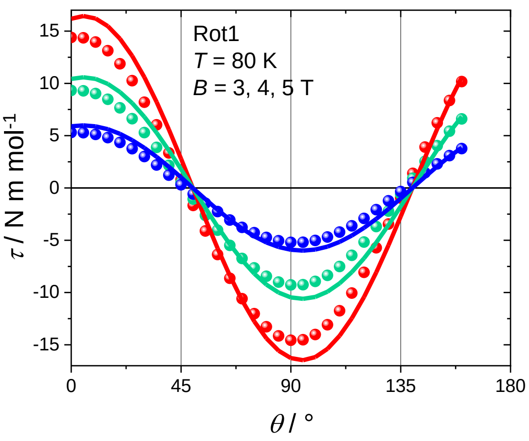
Rot1
T = 80 K
B = 3, 4, 5 T
τ / N m mol-1
θ / °

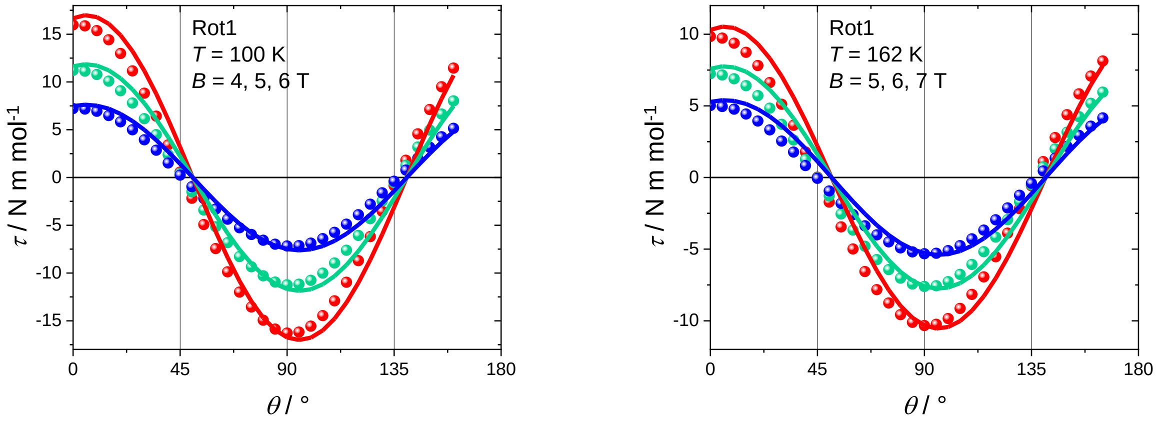

Figure S38. Torque curves obtained for **Er**: Rot1 (dots). A scaling factor has been applied to the experimental torque due to the incertitude on the crystal's mass.

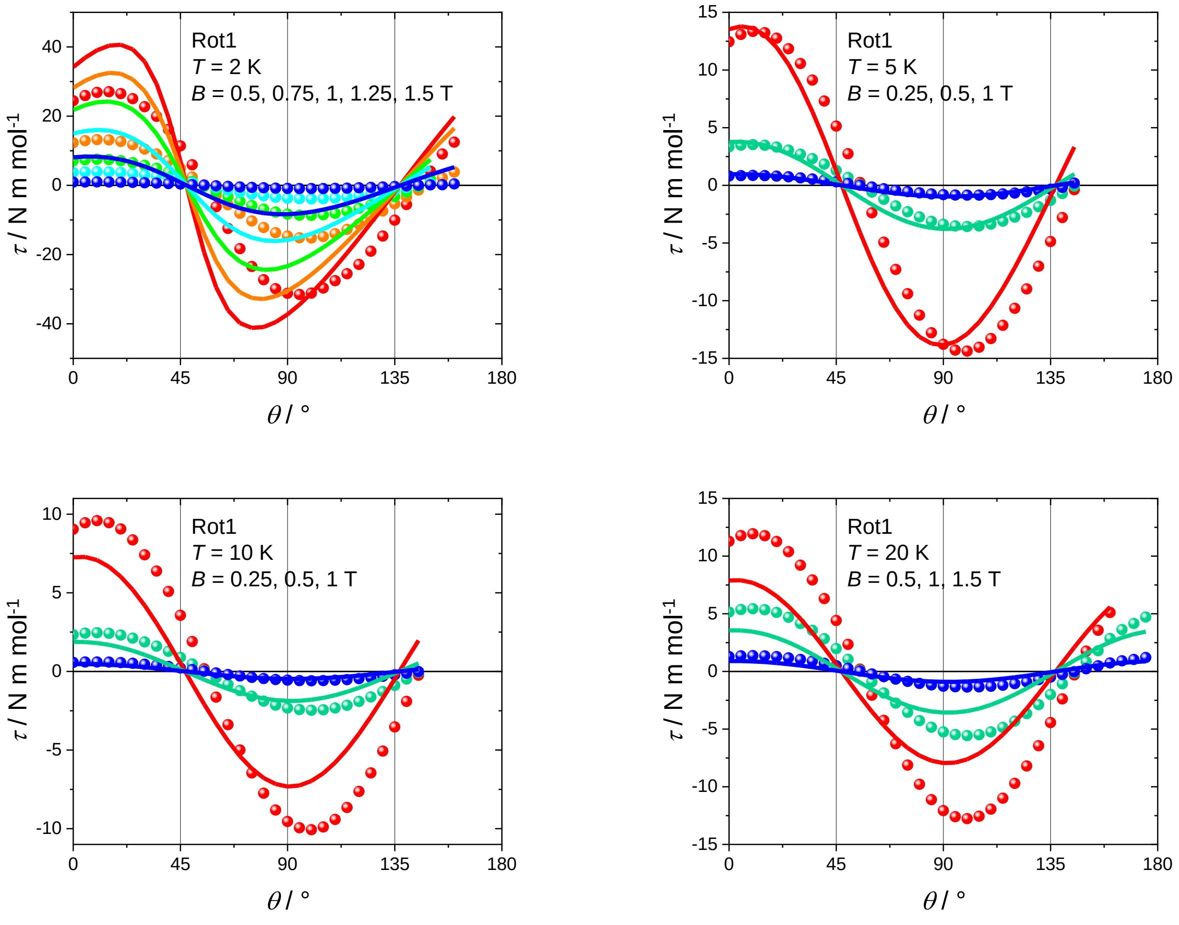

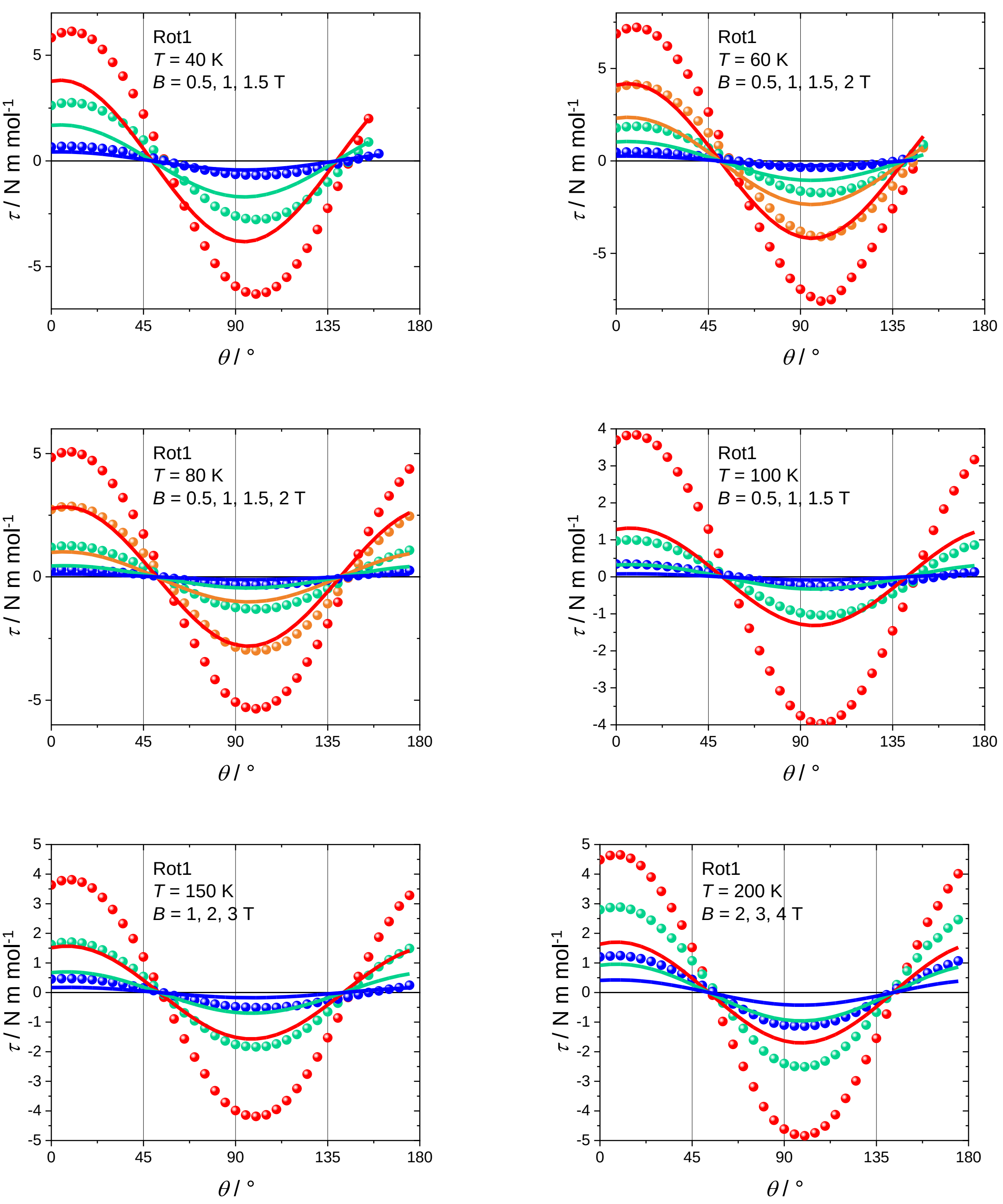

Rot1
T = 40 K
B = 0.5, 1, 1.5 T
Rot1
T = 60 K
B = 0.5, 1, 1.5, 2 T
Rot1
T = 80 K
B = 0.5, 1, 1.5, 2 T
Rot1
T = 100 K
B = 0.5, 1, 1.5 T
Rot1
T = 150 K
B = 1, 2, 3 T
Rot1
T = 200 K
B = 2, 3, 4 T
τ / N m mol-1
θ / °

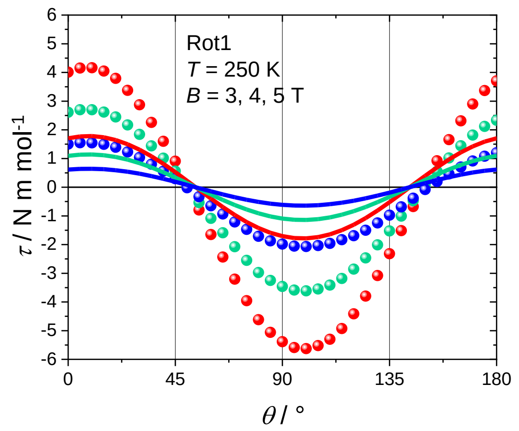

Figure S39. Torque curves obtained for **Tm**: Rot1 (dots). A scaling factor has been applied to the experimental torque due to the incertitude on the crystal's mass.

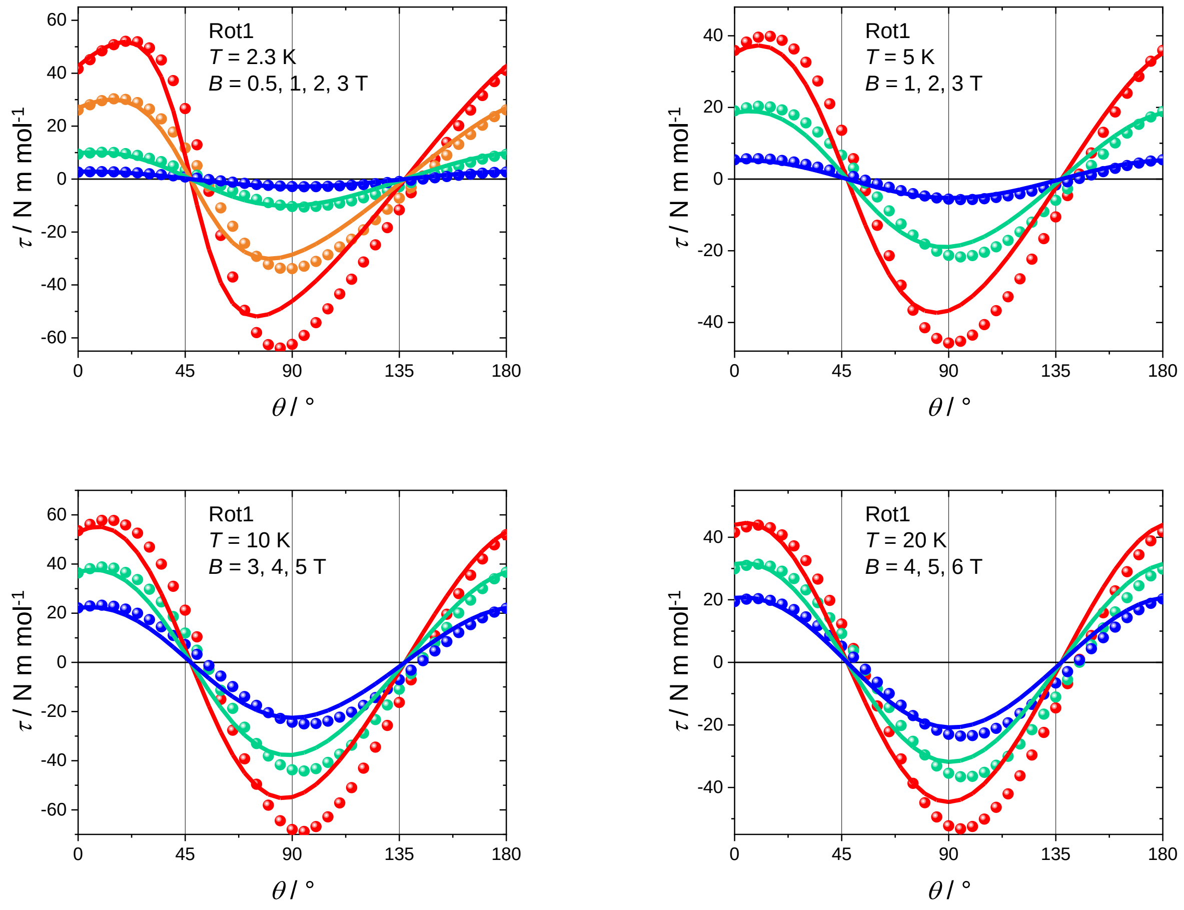

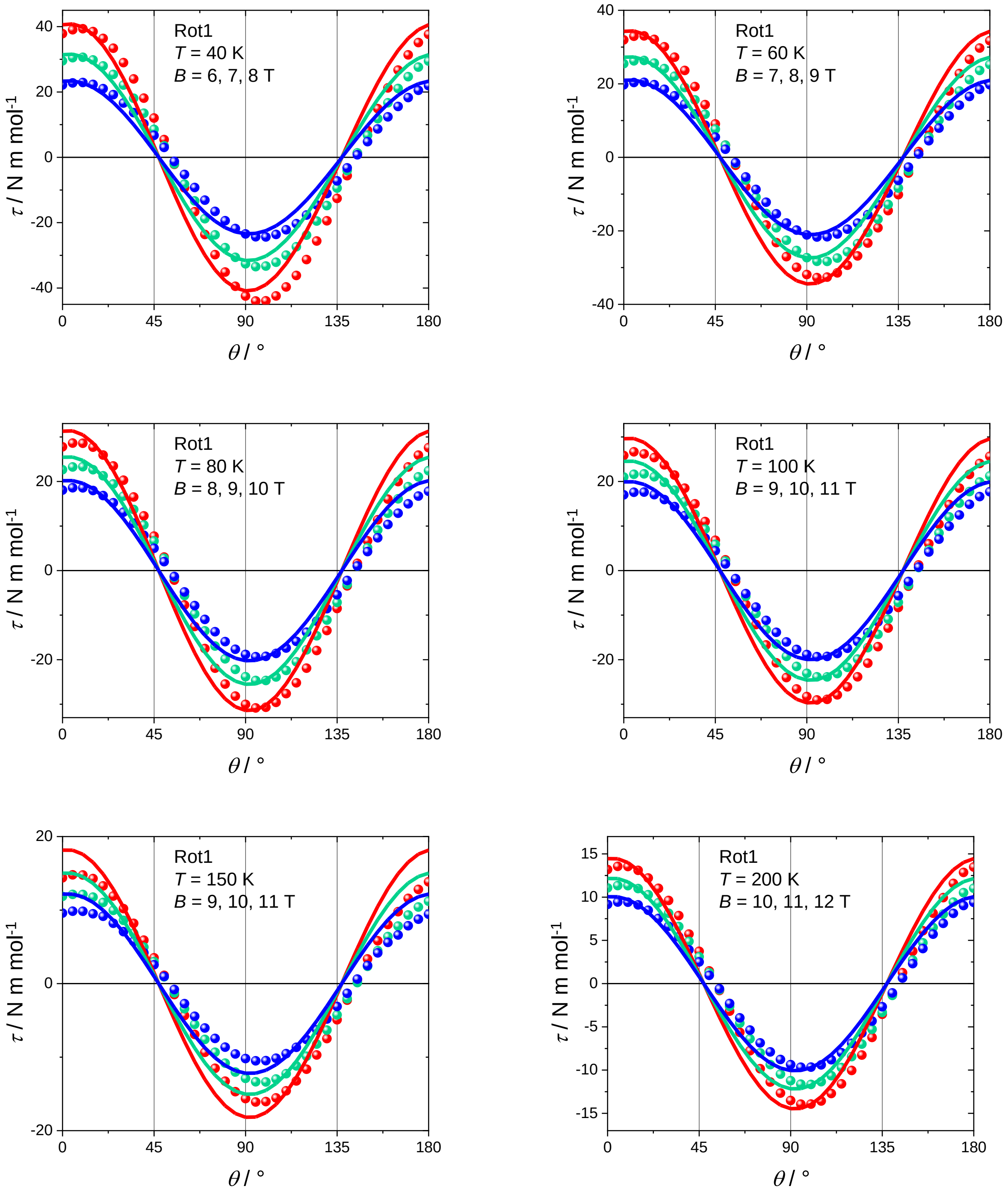

Figure S40. Torque curves obtained for **Yb**: Rot1 (dots). A scaling factor has been applied to the experimental torque due to the incertitude on the crystal's mass.

	Ce/Tb	Pr/Dy	Nd/Ho	Pm/Er	Sm/Tm	Eu/Yb
z	2	21	4	7	2	1
y	2	21	5	12	69	45
x	1	6	2	11	69	45

Table S12. Angle between the ab initio reference frame of the 6 couples differing by 7 f-electrons. All the values are expressed in degrees.

Other tables and Figures

		I Doublet	II Doublet
**Ce**	**Energy (cm^{-1})**	0	109
	**|±5/2 >**	0.2	9.4
	**|±3/2 >**	0.3	90.3
	**|±1/2 >**	99.5	0.3
**Nd**	**Energy (cm^{-1})**	0	81
	**|±9/2 >**	0.1	20.7
	**|±7/2 >**	0.2	2.9
	**|±5/2 >**	42.4	0.3
	**|±3/2 >**	56.3	0.9
	**|±1/2 >**	10.5	75.3
**Sm**	**Energy (cm^{-1})**	0	266
	**|±5/2 >**	95.1	4.4
	**|±3/2 >**	3.9	86.4
	**|±1/2 >**	1.1	9.2
**Dy**	**Energy (cm^{-1})**	0.00	47
	**|±15/2 >**	0.1	0.0
	**|±13/2 >**	0.4	0.5
	**|±11/2 >**	1.6	4.1
	**|±9/2 >**	4.7	2.0
	**|±7/2 >**	6.9	1.2
	**|±5/2 >**	9.0	14.5
	**|±3/2 >**	21.3	49.2
	**|±1/2 >**	56.1	28.1
**Er**	**Energy (cm^{-1})**	0	41
	**|±15/2 >**	20.3	59.5
	**|±13/2 >**	48.2	16.7
	**|±11/2 >**	11.8	2.6
	**|±9/2 >**	1.9	2.8
	**|±7/2 >**	5.9	4.7
	**|±5/2 >**	5.2	7.4
	**|±3/2 >**	3.9	4.8
	**|±1/2 >**	2.9	1.5
**Yb**	**Energy (cm^{-1})**	0.00	257
	**|±7/2 >**	96.8	0.2
	**|±5/2 >**	0.1	92.4
	**|±3/2 >**	0.1	7.4

	**|±1/2 >**	3.0	0.0

Table S13. Ab initio calculated energies and |m_j > percentage components of ground and first excited doublet states arising from the ground J multiplet of the **Ln** complexes with odd number of electrons (Kramers'ions). The quantization axis is along the Dy-O_{H2O} bond, i.e. the pseudo-tetragonal axis of the molecule.

		I	II	III	IV
**Pr**	**Energy (cm^{-1})**	0	112	145	235
	**|±4 >**	12.9	0.0	0.0	0.1
	**|±3 >**	0.1	11.0	10.5	0.4
	**|±2 >**	0.2	1.1	0.1	98.5
	**|±1 >**	0.2	87.7	89.3	0.9
	**|0 >**	86.6	0.2	0.0	0.0
**Pm**	**Energy (cm^{-1})**	0	41	65	74
	**|±4 >**	67.9	88.4	2.6	7.4
	**|±3 >**	0.1	2.2	83.6	32.5
	**|±2 >**	2.2	9.2	0.1	56.4
	**|±1 >**	1.3	0.2	13.1	3.6
	**|0 >**	28.5	0.1	0.4	0.1
**Tb**	**Energy (cm^{-1})**	0	2	11	15
	**|±6 >**	0.1	0.0	0.0	0.1
	**|±5 >**	0.0	1.7	1.5	0.1
	**|±4 >**	8.4	0.2	0.1	1.0
	**|±3 >**	0.5	13.9	15.9	0.0
	**|±2 >**	9.6	0.6	0.3	89.2
	**|±1 >**	1.1	82.7	82.1	0.5
	**|0 >**	80.4	0.9	0.1	8.7
**Ho**	**Energy (cm^{-1})**	0	8	25	45
	**|±8 >**	0.1	0.0	0.0	5.2
	**|±7 >**	0.0	3.6	4.3	0.0
	**|±6 >**	3.7	0.1	0.1	0.1
	**|±5 >**	0.2	0.2	1.1	0.9
	**|±4 >**	1.4	0.8	0.2	57.0
	**|±3 >**	0.5	58.6	55.5	0.4
	**|±2 >**	92.0	0.8	0.8	1.3
	**|±1 >**	1.2	35.9	36.8	0.7
	**|0 >**	0.8	0.0	1.2	34.4

Tm	**Energy (cm^{-1})**	0	2	193	198
	**|±6 >**	97.1	97.8	0.0	0.0
	**|±5 >**	0.0	0.0	93.3	94.8
	**|±4 >**	0.0	0.0	0.5	0.6
	**|±3 >**	0.0	0.0	0.3	0.2
	**|±2 >**	2.8	2.1	0.0	0.0
	**|±1 >**	0.0	0.0	5.8	4.3
	**|0 >**	0.0	0.0	0.0	0.0

Table S14. Ab initio calculated energies and $|m_j>$ percentage components of ground and first three excited singlet states arising from the ground J multiplet of the **Ln** complexes with even number of electrons. The quantization axis is along the Dy-O_{H2O} bond, i.e. the pseudo-tetragonal axis of the molecule. Plus and minus $|m_j>$ components have been summed up, as a consequence the sum along the row can go beyond 100% (up to 200%).

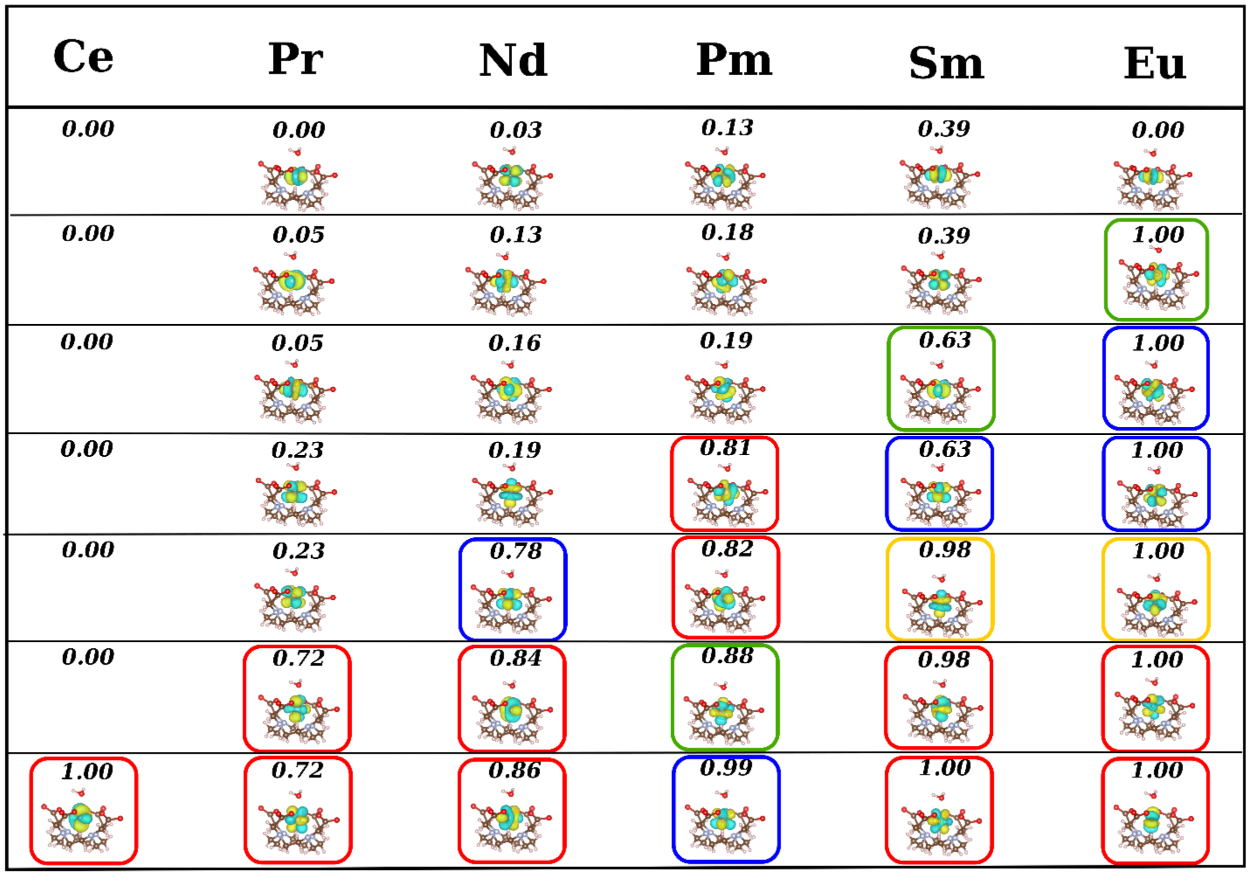

Ce	Pr	Nd	Pm	Sm	Eu
0.00	0.00	0.03	0.13	0.39	0.00
0.00	0.05	0.13	0.18	0.39	1.00
0.00	0.05	0.16	0.19	0.63	1.00
0.00	0.23	0.19	0.81	0.63	1.00
0.00	0.23	0.78	0.82	0.98	1.00
0.00	0.72	0.84	0.88	0.98	1.00
1.00	0.72	0.86	0.99	1.00	1.00

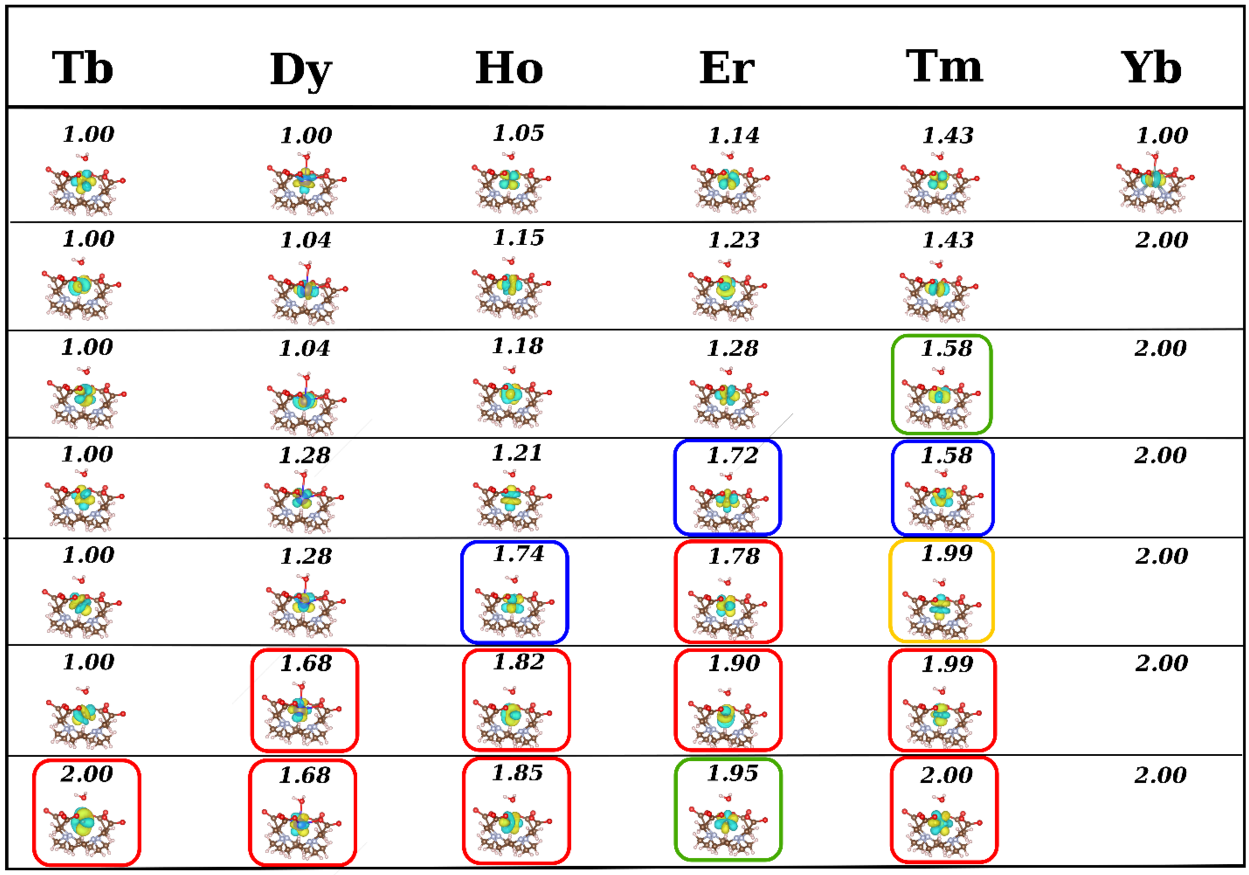

Tb	Dy	Ho	Er	Tm	Yb
1.00	1.00	1.05	1.14	1.43	1.00
1.00	1.04	1.15	1.23	1.43	2.00
1.00	1.04	1.18	1.28	1.58	2.00
1.00	1.28	1.21	1.72	1.58	2.00
1.00	1.28	1.74	1.78	1.99	2.00
1.00	1.68	1.82	1.90	1.99	2.00
2.00	1.68	1.85	1.95	2.00	2.00

Figure S41. 4f CASSCF/RASSI-SO orbitals for all the derivatives except Gd. 4f orbitals are associated with different m_l values, assuming the pseudo-C_4 axis of the complex as the quantization axis. The association between colours of the squares and m_l values is the following: red/m_l=1, blue/m_l=2, green/m_l=3, yellow/m_l=0. Squares are around the occupied orbitals in the first half of the series, and around the doubly occupied ones in the second half.

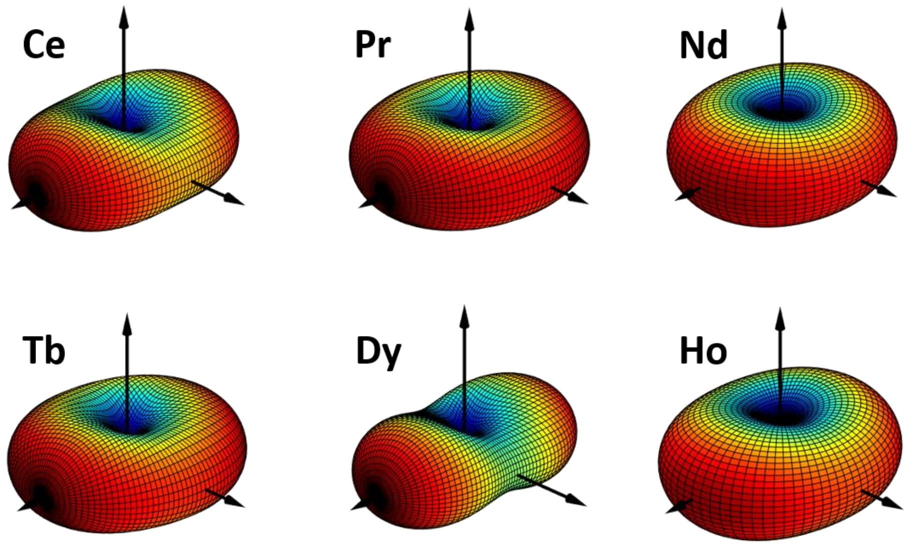

Figure S42. Magnetic susceptibility tensor of **Ce**, **Pr**, **Nd**, **Tb**, **Dy** and **Ho** calculated at *T* = 100 K and *B* = 0.1 T.

	χ_{xx}	χ_{yy}	χ_{zz}
**Ce**	0.120008	0.780343	1.022949
**Pr**	0.171815	1.306970	1.672415
**Nd**	0.427485	1.411817	1.672415
**Pm**	0.517768	0.535366	1.127166
**Sm**	0.091090	0.158643	0.160567
**Eu**	0.553464	0.579252	2.069784
**Gd**	7.820149	7.860695	7.864156
**Tb**	2.311098	14.784955	17.020447
**Dy**	2.462428	13.842541	24.348631
**Ho**	5.013363	15.934346	18.603591
**Er**	7.281062	7.515136	17.645502
**Tm**	1.308370	1.339802	17.162728
**Yb**	0.461581	0.545607	5.533711

Table S15. Principal values of the susceptibility tensors ($cm^3\,mol^{-1}$) calculated at *T* = 100 K. The *z* axis corresponds to the highest susceptibility axis for an easy comparison.

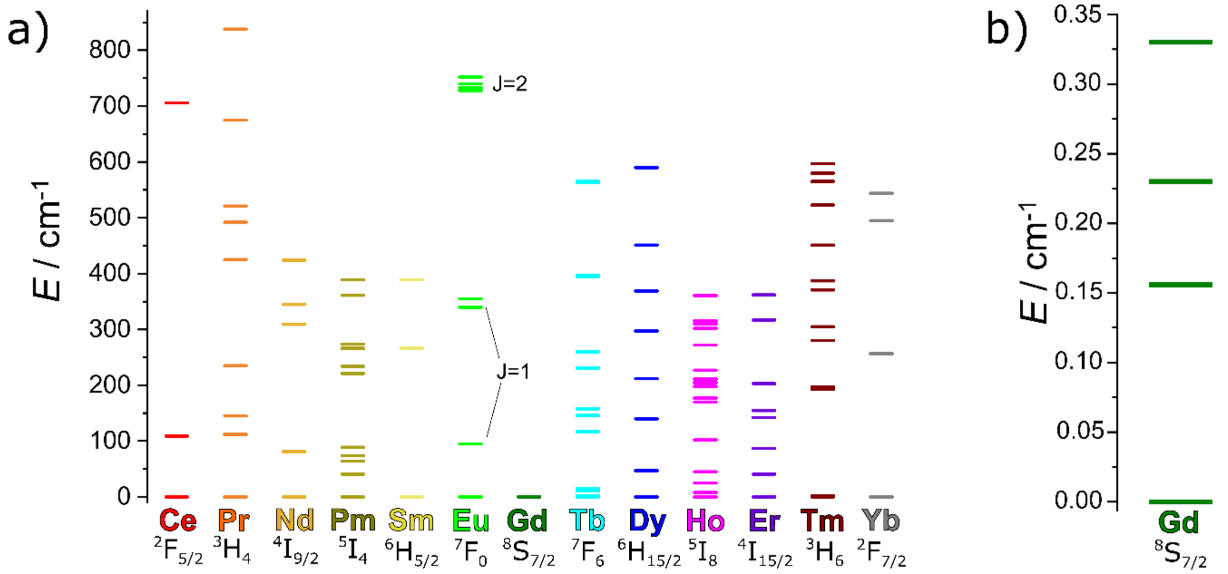

Figure S43. Ab initio computed electronic structure of all the derivatives.

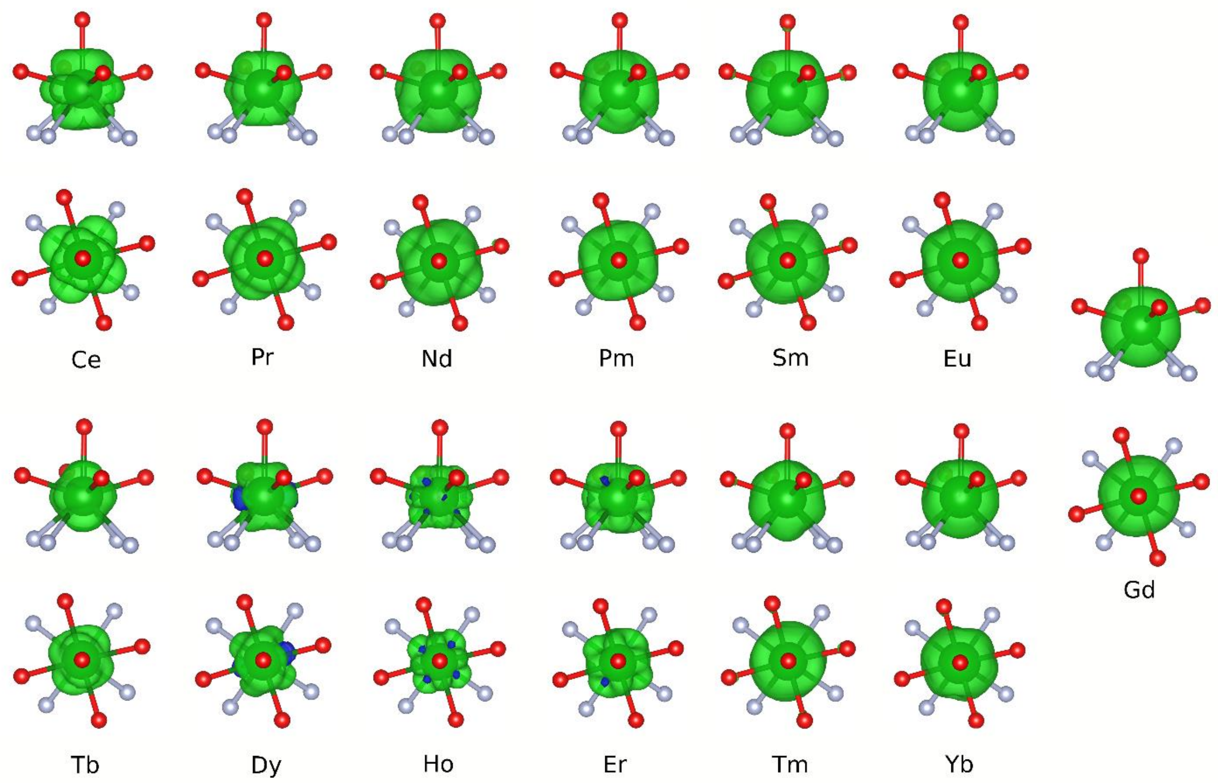

Figure S44. Electron density of the electrons in the 4*f* orbitals (contour value $5 \cdot 10^{-4}$ e^-/a_0^3). The shown density for **Tb-Yb** was obtained subtracting the **Gd** electron density, computed within each corresponding molecular geometry, to highlight the similarity between the $4f^{\,n}$ and the $4f^{\,n+7}$ ions. The negative electron density (blue areas) is an artefact that arises from such subtraction.

References:

1. Car, P. E.; Perfetti, M.; Mannini, M.; Favre, A.; Caneschi, A.; Sessoli, R., Giant field dependence of the low temperature relaxation of the magnetization in a dysprosium(III)-DOTA complex. *Chem. Commun.* **2011,** *47* (13), 3751-3753.
2. Stoll, S.; Schweiger, A., EasySpin, a comprehensive software package for spectral simulation and analysis in EPR. *J. Magn. Reson.* **2006,** *178* (1), 42-55.
3. Rudowicz, C.; Chung, C., The generalization of the extended Stevens operators to higher ranks and spins, and a systematic review of the tables of the tensor operators and their matrix elements. *J. Phys. Condens. Matter* **2004,** *16* (32), 5825.
4. Boulon, M. E.; Cucinotta, G.; Luzon, J.; Degl'Innocenti, C.; Perfetti, M.; Bernot, K.; Calvez, G.; Caneschi, A.; Sessoli, R., Magnetic Anisotropy and Spin-Parity Effect Along the Series of Lanthanide Complexes with DOTA. *Angew. Chem. Int. Ed.* **2013,** *125* (1), 368-372.
5. Boulon, M. E.; Cucinotta, G.; Luzon, J.; Degl'Innocenti, C.; Perfetti, M.; Bernot, K.; Calvez, G.; Caneschi, A.; Sessoli, R., Magnetic Anisotropy and Spin-Parity Effect Along the Series of Lanthanide Complexes with DOTA. *Angew. Chem.* **2013,** *125* (1), 368-372.
6. Briganti, M.; Garcia, G. F.; Jung, J.; Sessoli, R.; Le Guennic, B.; Totti, F., Covalency and magnetic anisotropy in Lanthanide Single Mole-cule Magnets: the DyDOTA Archetype. *Chem. Sci.* **2019,** *10*, 7233-7245.
7. Cucinotta, G.; Perfetti, M.; Luzon, J.; Etienne, M.; Car, P. E.; Caneschi, A.; Calvez, G.; Bernot, K.; Sessoli, R., Magnetic Anisotropy in a Dysprosium/DOTA Single-Molecule Magnet: Beyond Simple Magneto-Structural Correlations. *Angew. Chem. Int. Ed.* **2012,** *51* (7), 1606-1610.
8. Bernot, K.; Luzon, J.; Bogani, L.; Etienne, M.; Sangregorio, C.; Shanmugam, M.; Caneschi, A.; Sessoli, R.; Gatteschi, D., Magnetic Anisotropy of Dysprosium(III) in a Low-Symmetry Environment: a Theoretical and Experimental Investigation. *J. Am. Chem. Soc.* **2009,** *131* (15), 5573-5579.
